# Recent Advances in the Application of Nanocatalysts for the Hydrodeoxygenation of Lignin and Its Derivatives to Jet-Fuel-Range Cycloalkanes and Aromatics

**DOI:** 10.3390/bioengineering13101218

**Published:** 2026-10-19

**Authors:** Kexin Gu, Dongyu Zhang, Meiting Yin, Shu Yan, Wenjun Fang, Yongsheng Guo

**Affiliations:** 1 Department of Chemistry, Zhejiang University, Hangzhou 310058, China; 12537019@zju.edu.cn (K.G.); 12537073@zju.edu.cn (D.Z.); 22437027@zju.edu.cn (M.Y.); fwjun@zju.edu.cn (W.F.); 2 Center of Chemistry for Frontier Technologies, Zhejiang University, Hangzhou 310058, China; 3 Zhejiang Key Laboratory of Low-Carbon Synthesis of Value-Added Chemicals, Zhejiang University, Hangzhou 310058, China

**Keywords:** lignin, hydrodeoxygenation, nanocatalysis, sustainable aviation fuel (SAF)

## Abstract

Lignin is the sole renewable organic carbon resource that is intrinsically rich in aromatic rings. Its catalytic hydrodeoxygenation (HDO) to produce jet-fuel-range cycloalkanes and aromatics represents a pivotal route toward achieving sustainable aviation fuel. The central challenge of HDO lies in achieving selective C-O bond cleavage while precisely controlling the hydrogenation of aromatic rings. This review systematically summarizes recent advances in nanocatalysts for this application, covering molybdenum-based catalysts (edge-site engineering and oxygen vacancy regulation in MoS_2_), nickel-based catalysts (optimization of low-temperature activity in nickel phosphide and hydrogen spillover strategies), bimetallic catalysts (geometric/electronic/stabilization/synergistic effects), and bifunctional catalysts (synergy between acid sites and metal sites), with particular emphasis on the emerging single-atom catalysts (coordination environment and charge density regulation). The functional roles of nanocatalysts in cascade conversion processes are also discussed. Finally, the future directions for the rational design of highly efficient nanocatalytic systems are outlined, aiming to provide insights for the targeted synthesis of lignin-based jet-fuel-range cycloalkanes and aromatics.

## 1. Introduction

**Figure 1:**
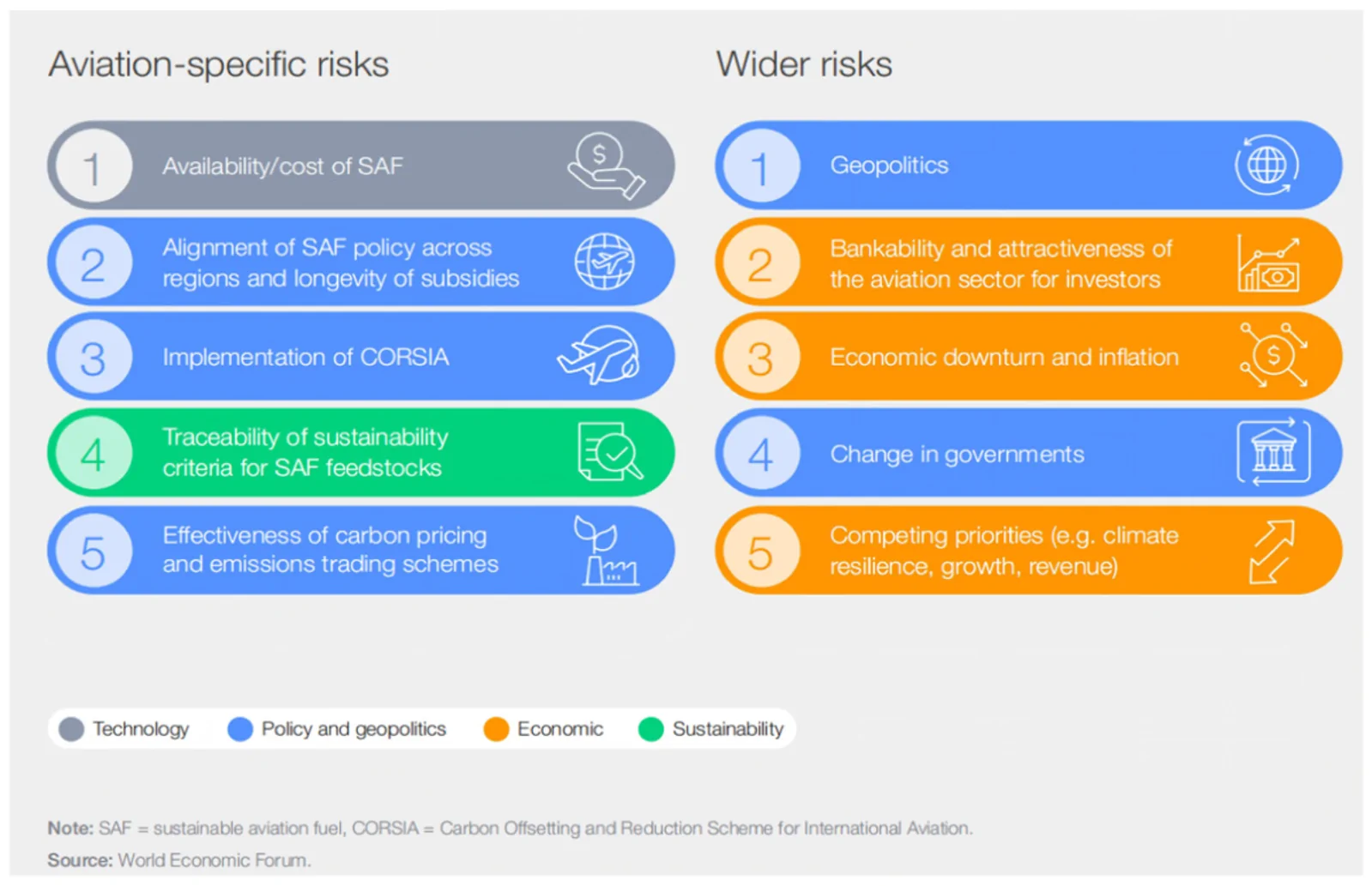
The top ten challenges impeding the decarbonization of aviation. Reproduced with permission from [7].

Biomass is defined as any organic matter that is regenerable on a timescale relevant to human use. Among various renewable resources (such as solar energy and wind power, etc.), biomass stands out as the sole naturally occurring renewable organic carbon resource, conferring a unique advantage in producing high-value-added products [1,2,3,4]. In the energy sector, amid pressing challenges such as the energy crisis and climate deterioration, biomass holds a significant position in the context of energy transition and the “dual carbon” goals. Globally, the transportation sector currently accounts for approximately one-quarter of energy-related CO_2_ emissions, with aviation and shipping together contributing about 4–5%. Within the aviation sector, sustainable aviation fuel (SAF) accounts for only 0.1% [5]. SAF represents a practical and immediately deployable solution for aviation decarbonization, as its life-cycle CO_2_ emissions can be reduced by up to 80% compared to conventional jet fuel [6]. The World Economic Forum’s 2025 Global Aviation Sustainability Outlook particularly emphasizes the critical role of SAF in advancing aviation decarbonization, and identifies the availability and cost of SAF as one of the top ten challenges impeding this process (Figure 1) [7]. Therefore, the importance of developing biomass-derived jet fuel is self-evident.

Conventional aviation jet fuel is composed of paraffins, cycloalkanes and aromatics, with a carbon number range of approximately C9 to C16 [8,9]. Among these, cycloalkanes exhibit higher thermal stability, greater energy density, and shorter ignition delay time compared to *n*-alkanes [10,11,12,13]. Additionally, the favorable low-temperature properties of cycloalkanes help maintain fuel fluidity at low temperatures while preserving energy density [10]. Natelson et al. [14] demonstrated that alkyl-substituted cyclohexanes generate various unsaturated alkyl radicals and cycloalkenes during low-temperature combustion decomposition pathways, thereby conferring two-stage ignition capability. Aromatic hydrocarbons, despite their inferior thermal oxidative stability, play a crucial role in determining the fuel system safety, lubricity, and the swelling of elastomeric O-ring seals in fuel tanks [15]. Studies have indicated low-molecular-weight alkylbenzenes as ideal aromatic components for fully synthetic aviation turbine fuels [8,16]. Furthermore, monocyclic aromatics exhibit advantages over polycyclic aromatics in terms of heat release profile and emissions performance [16,17].

However, the current mainstream technical routes for producing jet fuel from biomass components are each confronted with a distinct set of challenges: the biomass gasification–Fischer–Tropsch (FT) synthesis hydroprocessing route suffers from limited and unstable feedstock supply, as well as high processing costs [18]. The hydroprocessed esters and fatty acids (HEFA) route, which relies on vegetable oils and animal fats, directly competes with the food supply chain, and is further plagued by catalyst deactivation, high production costs, and relatively higher carbon emissions [19,20]. The alcohol-to-jet (ATJ) route, based on bio-derived light alcohols, faces issues such as complex and multi-step processes, high energy intensity, low Technology Readiness Level (TRL), and carbon emissions that are significantly higher than those of other mainstream SAF pathways [21]. The power-to-liquid (PtL/e-fuels) route encounters challenges that primarily originate from the upstream electrolysis stage, including high capital costs, low energy conversion efficiency with a substantial portion of electrical energy being dissipated as heat, and a similarly low TRL [22,23]. Furthermore, a common shortcoming across these routes is that the resulting products are predominantly paraffinic hydrocarbons, with insufficient contents of cycloalkanes and aromatics to meet fuel specifications, thereby necessitating blending with conventional petroleum-based jet fuel [24]. Relevant studies suggest that the blending ratio of such alternative fuels should not exceed 50%, and that the volume fraction of aromatics should not fall below 8% [25,26].

**Figure 2:**
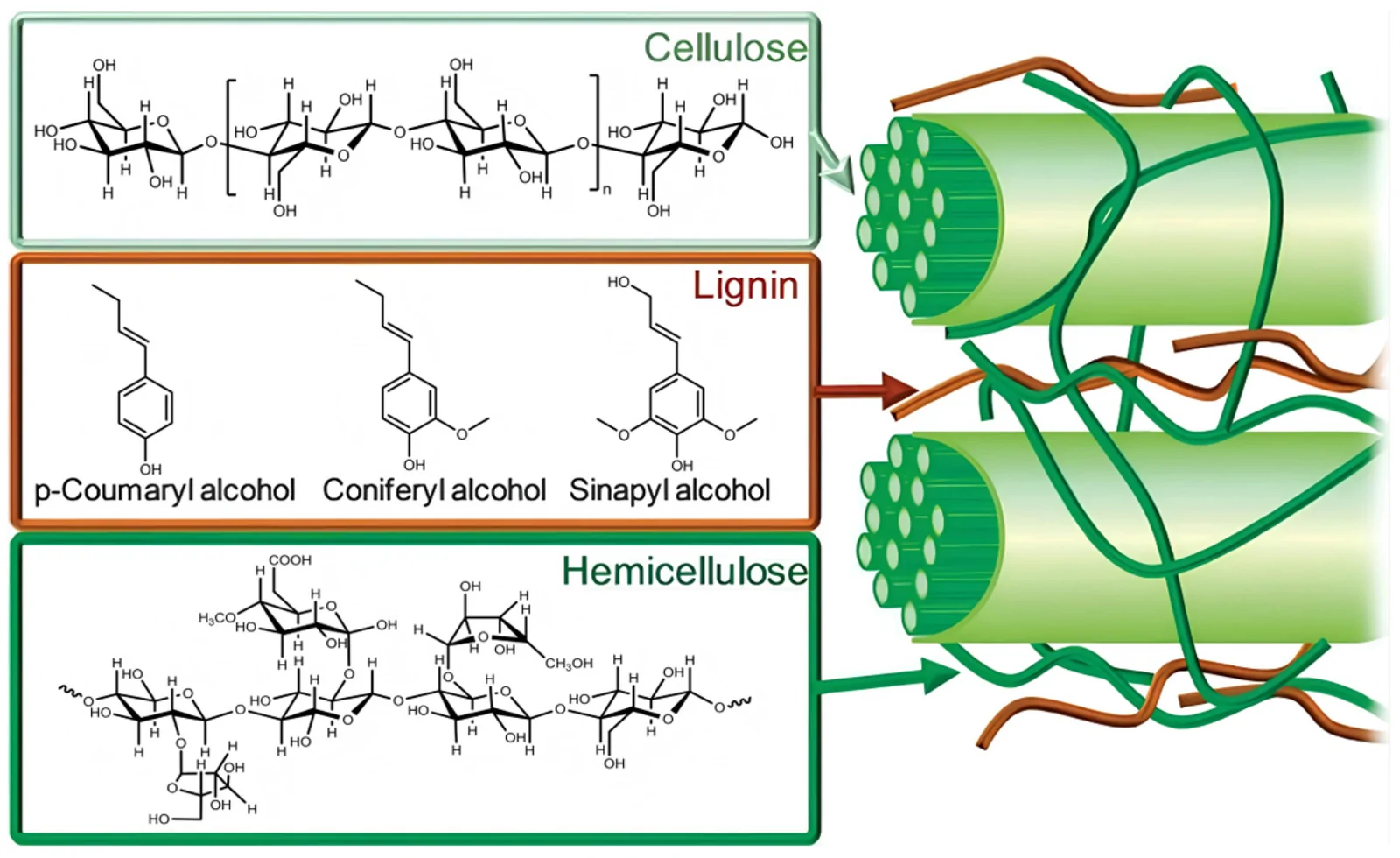
The structure of cellulose, hemicellulose and lignin in woody cellulose. Reproduced with permission from [28]; published by Royal Society of Chemistry, 2012.

In contrast, the lignocellulosic biomass conversion (LCBC) route, which utilizes lignocellulosic biomass, including lignin, as feedstock, offers inherent advantages for producing SAF: 1. Abundant and underutilized feedstock. The global annual production of lignocellulosic biomass is approximately 182 billion tonnes, of which only about 8 billion tonnes are currently utilized [27]. Lignocellulose mainly consists of three components: hemicellulose, cellulose, and lignin (Figure 2) [28]. Lignin accounts for about 15–35% of the total mass and up to 40% of the total energy content [3]. Moreover, lignin is non-edible, and its use does not compete directly with the food supply chain; it is therefore recognized as a promising alternative to fossil-based fuels and chemicals [1]. 2. Intrinsic structural compatibility with jet-fuel aromatic and cycloalkane fractions. Lignin is the only naturally occurring polymer that is rich in aromatic rings, which endows it with an intrinsic structural advantage for “naturally matching” the aromatic and cycloalkane fractions required in jet fuel [28,29]. This inherent compatibility provides a feasible pathway toward developing fully drop-in jet fuel that can be used at 100% without blending limitations [24]. 3. Superior carbon footprint. The LCBC route can achieve up to 94% greenhouse gas emission reduction over its life cycle, significantly outperforming the HEFA route and the PtL route in terms of carbon performance [20].

**Figure 3:**
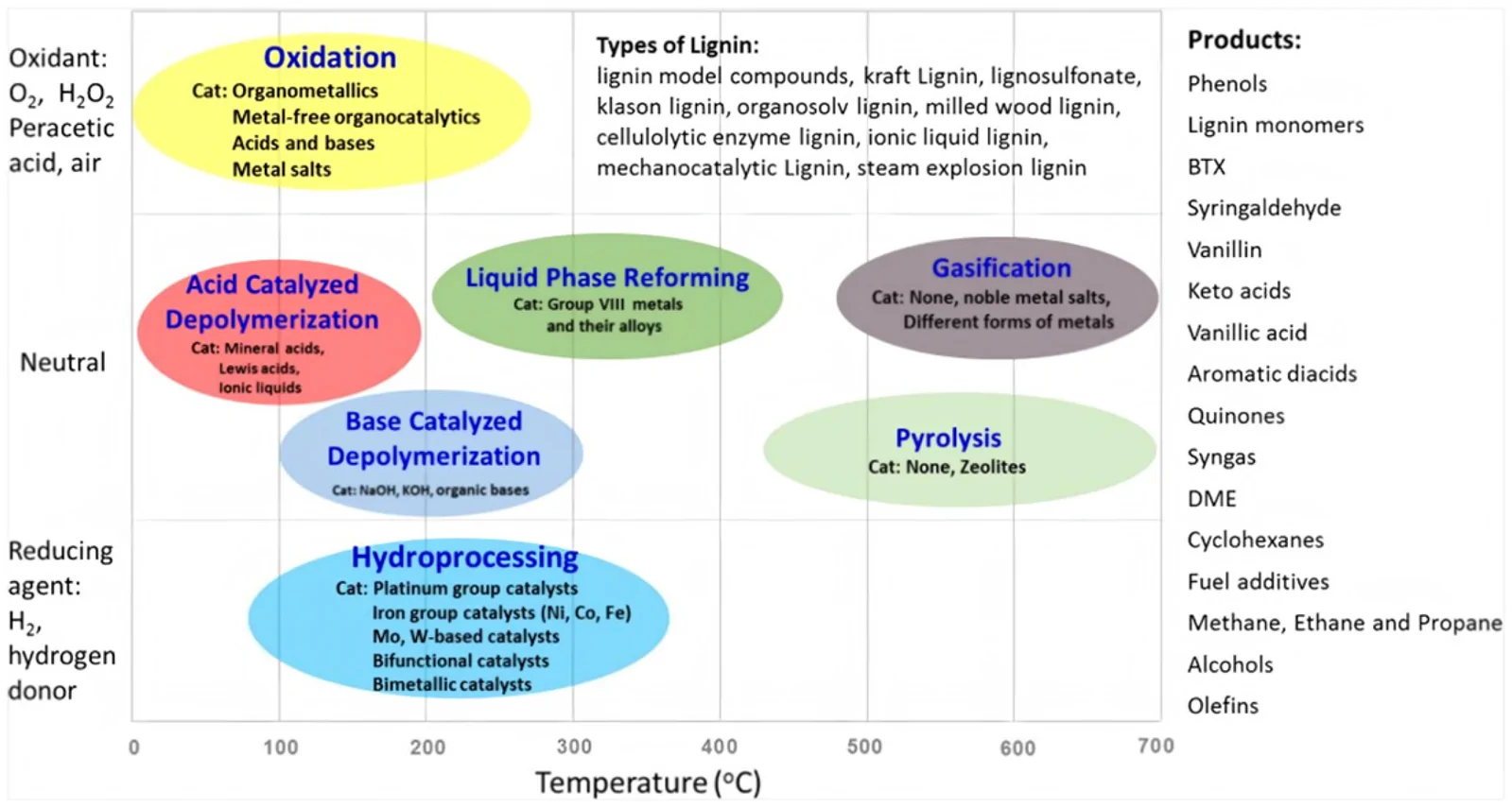
Summary of the lignin conversion process. Reproduced with permission from [1]; published by American Chemical Society, 2015.

Although the composition and content of lignin vary among different plant species, it is generally composed of three basic C9 structural units: p-coumaryl alcohol (H), coniferyl alcohol (G), and sinapyl alcohol (S). These units are linked through ether bonds (C-O-C) and carbon–carbon bonds (C-C) (Figure 2) [28,29]. In native lignin, ether bonds account for two-thirds or more of the total linkages. Lignin can be depolymerized via various approaches, including pyrolysis [30], gasification [31,32,33], hydrogenolysis [34,35,36], oxidation [37], as well as photo-/electrocatalytic degradation [38,39,40], microwave-assisted degradation [41,42,43], or enzymatic hydrolysis [44,45] (Figure 3) [1]. The primary products are oxygenated aromatic compounds, which cannot be directly utilized as jet fuel. Among the various upgrading strategies, hydrodeoxygenation (HDO) is recognized as the most effective approach for upgrading lignin-derived oils [1,46]. The HDO process proceeds via two parallel reaction pathways: direct deoxygenation (DDO) and hydrogenation–deoxygenation (HYD). In the DDO pathway, the aromatic ring structure is preserved through hydrogenolysis of the C-O bonds in the side chains, yielding aromatics as the main products. In the HYD pathway, the aromatic ring is first hydrogenated to saturation, followed by hydrogenolysis of the C-O bonds in the side chains, producing cycloalkanes such as cyclohexane and propylcyclohexane [47], which are key components of aviation kerosene. However, direct HDO of lignin-derived monomers typically yields lower-molecular-weight aromatics and cycloalkanes such as benzene (C6), toluene (C7), cyclohexane (C6), and methylcyclohexane (C7). Although these compounds fall below the ideal jet-fuel carbon range, they can be upgraded to jet-fuel-range hydrocarbons through C-C coupling reactions such as alkylation or aldol condensation, followed by HDO [48,49]. Therefore, in this review, the term “jet-fuel-range products” is used broadly to denote hydrocarbons that can ultimately be converted into jet fuel components, either directly or after appropriate C-C coupling/upgrading steps. We do not strictly distinguish C6-C8 and C9-C16 products in the subsequent discussion. Instead, we focus on the catalytic strategies that enable selective C-O cleavage and controlled hydrogenation, with the understanding that low-molecular-weight products can be further upgraded to meet jet fuel specifications.

However, achieving the desired selectivity control in the aforementioned pathways remains a formidable challenge. The HDO reaction involves competing C-O bond cleavage and aromatic ring hydrogenation, which impose markedly different requirements on the geometric configuration and electronic density of the catalyst active sites [50]. Catalysts developed for lignin HDO include noble metals, non-noble metals, and their nitrides, carbides, and phosphides. Studies have demonstrated that nanocatalysts, in which the active sites or active phases are controlled at the nanoscale (typically 1–100 nm), exhibit unique reactivity in lignin HDO reactions [51]. Taking metal catalysts as an example, nanoscale metal particles (typically < 5 nm) expose a high density of unsaturated coordination sites, with a significantly increased proportion of surface steps and edge sites, which can substantially lower the energy barrier for C-O bond cleavage [52,53]. Mondelli et al. [52], from the perspective of fundamental catalyst design, pointed out that the particle size and chemical state of the metal played a decisive role in determining the activity, selectivity, and stability of the catalyst. Owing to the unique structure-sensitive nature of competing reactions, tuning the size of metal nanoparticles can effectively modulate the product distribution in many hydrogenation, oxidation, and reforming reactions. Moreover, smaller particle sizes also imply a higher density of accessible surface metal atoms, which enhances the catalytic activity for both C-O bond cleavage and hydrogenation, highlighting the importance of precisely tailoring the particle size of metal and metal–support interactions for achieving high target product selectivity and catalytic efficiency in lignin valorization. In fact, the strong metal–support interaction (SMSI) is a size-dependent phenomenon: the interfacial contact area and the extent of charge transfer between metal and support are governed by the dimensions of the active phase, endowing it with a critical role in enhancing the reactivity and stability of the catalyst [54,55]. In addition to geometric and interfacial effects, electronic and defect-related effects are likewise intimately tied to the nanoscale and play a decisive role in catalysis. When metal particles are reduced to the nanoscale, the proportion of surface atoms increases dramatically, and the electronic structure undergoes substantial changes, including shifts in the d-band center, alterations in oxidation state, and quantum size effects, which in turn determine adsorption energies and reaction barriers [51,56]. Similarly, defect chemistry, including oxygen vacancies, coordinatively unsaturated sites, and cationic vacancies, is also intimately linked to nanoscale structure, as the formation and stabilization of these defects depend on particle size, morphology, and surface reconstruction [57]. Begum et al. [58] demonstrated that the particle size of Ru significantly influenced its catalytic performance in the HDO of lignin-derived compounds: specifically, undercoordinated Ru sites were more favorable for C-O bond cleavage, whereas ring hydrogenation occurred preferentially on coordinatively saturated sites.

This review systematically summarizes recent research progress on nanocatalysts for the HDO of lignin to produce jet-fuel-range cycloalkanes and aromatics. First, molybdenum-based catalysts are introduced, with a focus on edge-site engineering of MoS_2_ and strategies for activating inert basal planes, oxygen vacancy regulation in molybdenum oxides, and the noble-metal-like catalytic behavior of molybdenum carbides and nitrides. Second, nickel-based catalysts are discussed, emphasizing hydrogen spillover and transfer hydrogenation strategies, as well as activity modulation in C-O/C-C bond cleavage. Third, the metal–metal interaction (geometric, electronic, stabilization, and synergistic effects) of bimetallic catalysts and their influence on deoxygenation selectivity are explored. Subsequently, bifunctional catalysts (including metal–zeolite, metal–metal oxide, and metal–polyoxometalate composite systems) are highlighted, elucidating the synergistic mechanisms between acid sites and metal active sites in cascade deoxygenation, isomerization, and selectivity control. Furthermore, emerging single-atom catalysts are reviewed for their cutting-edge achievements to achieve precise product selectivity via fine-tuning of electronic structure. Finally, the current challenges are summarized, and future directions for the rational design of high-performance nanocatalytic systems are envisioned, with the aim of providing theoretical foundations and technical references for the targeted synthesis of aromatic and cycloalkane components in lignin-based SAF.

## 2. Nanocatalysts

Platinum-group metals (PGMs, such as Ru, Rh, Pd, and Pt) and certain non-noble metals (e.g., Mo, Ni, and Co) possess excellent hydrogenation and deoxygenation capabilities and have been widely used in the HDO of bio-oil [59,60,61,62]. However, the use of PGMs alone as catalysts is not an optimal choice for HDO processes [63,64,65], owing to their high cost and their excessively strong hydrogenation activity, which readily saturates aromatic rings and thereby compromises the retention of the aromatic fraction required in jet fuel. Consequently, PGMs are often employed supported on functional supports, such as zeolites, metal oxides, and polyoxometalates, etc. [1], a strategy that will be elaborated in Section 2.4 (Bifunctional Catalysts). In contrast, non-noble metals like Mo and Ni are abundant and cost-effective. Nevertheless, these catalysts typically suffer from issues such as partial hydrogenation and a high propensity for coke deposition, necessitating performance modulation through approaches such as facet engineering or heteroatom doping. Furthermore, it should be emphasized that, although some of the studies cited below also examine lignin-derived oil or raw/technical lignin, mechanistic work in this field has largely relied on model compounds. This is a reasonable starting point: model compounds allow specific C-O linkages and reaction pathways to be isolated, from which broader design principles can be distilled to guide the rational design of future catalysts. By contrast, studies on the HDO of real lignin remain relatively scarce and less mature. Reaction conditions are often incompletely reported, and performance metrics vary widely across studies. As a result, the data compiled in the table are difficult to compare and interpret consistently. For this reason, the table includes only data for lignin model compounds, while real-lignin studies are discussed in more detail in Section 3.

### 2.1. Molybdenum-Based Catalysts

**Figure 4:**
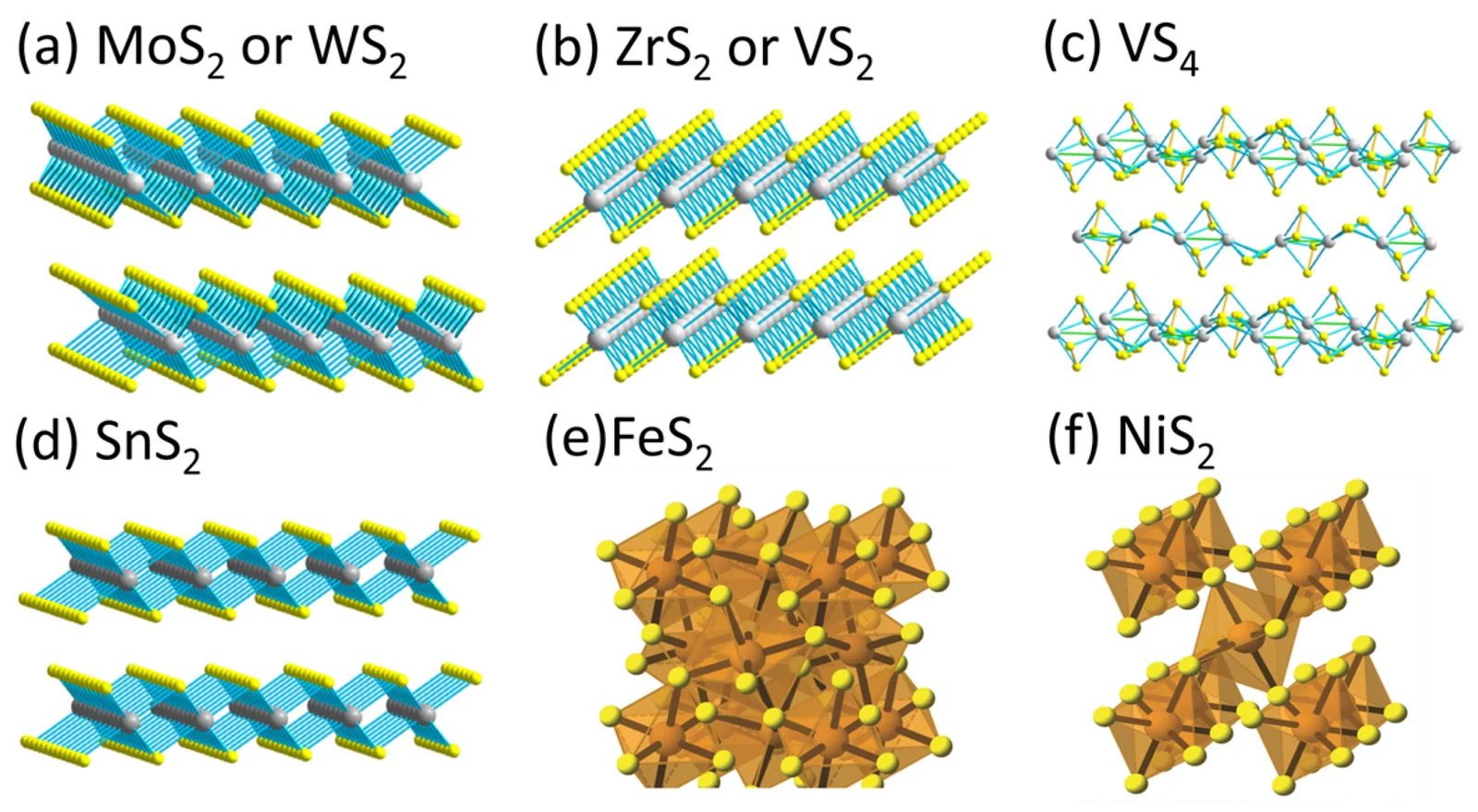
Schematic diagram of the three-dimensional structures of selected transition metal sulfides. (**a**–**d**) Layered structures. Reproduced with permission from [78]; published by RSC Publishing, 2013. (**e**,**f**) Non-layered structures. Reproduced with permission from [79]; published by Elsevier, 2014.

**Figure 5:**
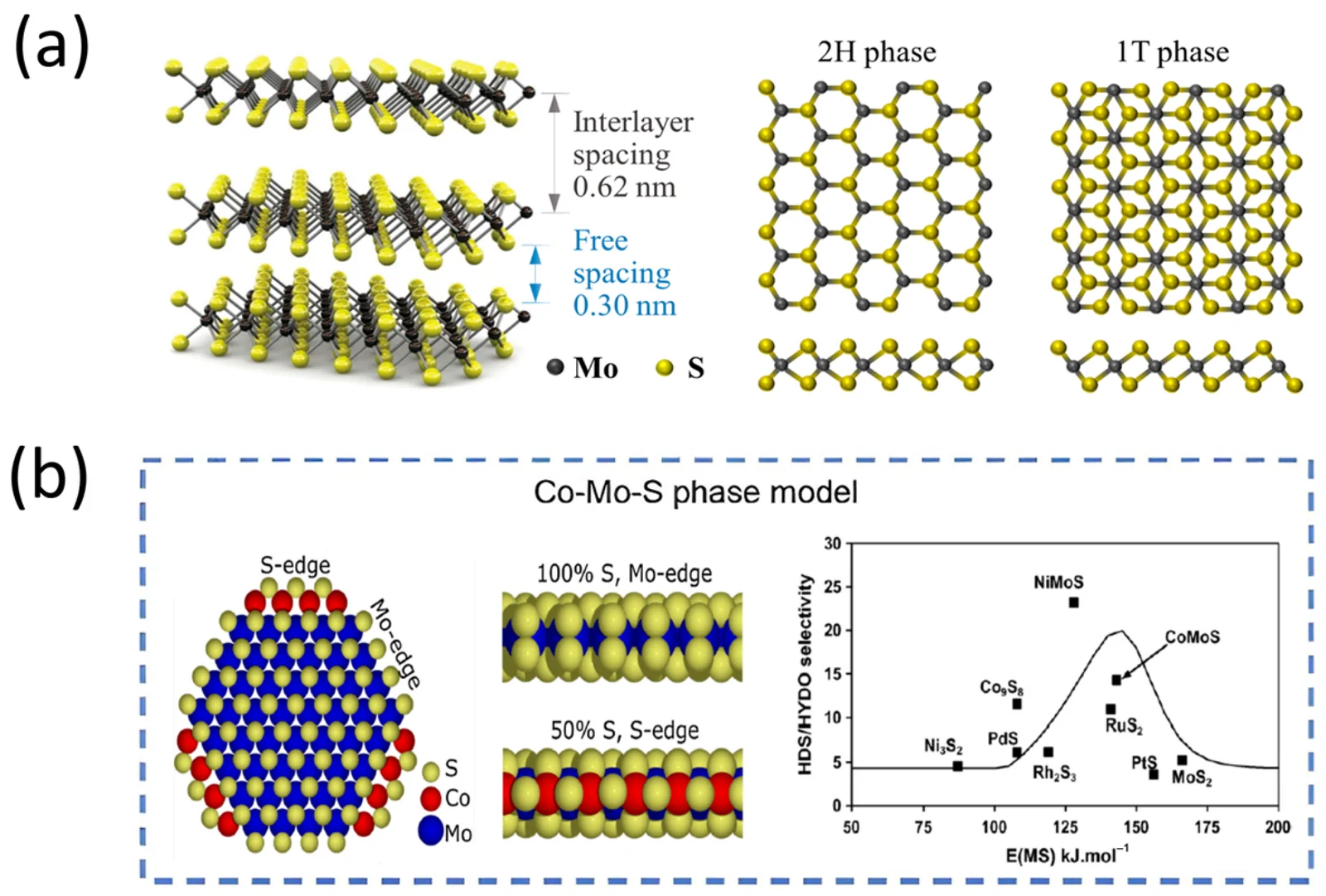
(**a**) MoS_2_ structure and HDO reaction mechanism of p-cresol on MoS_2_. Reproduced with permission from [80]; published by American Chemical Society, 2017. (**b**) Co-Mo-S active phase model. Reproduced with permission from [81]; published by Elsevier, 2008. Reproduced with permission from [82]; published by Elsevier, 2016.

Molybdenum-based catalysts include sulfides, oxides, nitrides, carbides, and others, and have been studied since as early as the 1980s, with molybdenum sulfides being the most extensively studied among them [66]. Layered MoS_2_ is currently one of the most widely reported transition metal sulfide catalysts for the HDO of lignin (Figure 4). For MoS_2_ (Figure 5a), the dominant exposed planes are predominantly the inert basal planes, while the density of active edge sites is governed by the extent of the edge planes. The number of stacked layers is a crucial factor in increasing the edge plane surface area [67,68,69,70]. Therefore, maximizing the exposure of edge sites through controlled growth of MoS_2_ nanostructures represents a viable strategy for enhancing the catalytic activity of MoS_2_-based catalysts. Table 1 summarizes recent studies on the application of molybdenum-based catalysts in the HDO of lignin and its derivatives.

**Table 1:** A summary of recent literature on the application of molybdenum-based catalysts in the catalytic HDO of lignin.

Year	Catalyst	Substrate	Major Product	Catalyst to Biomass Ratio (C:B)	Size (nm)	Reaction Conditions				Conv. (%)	Sel. (%)	Ref.
						T (°C)	P (MPa)	Time (h)	Solvent			
2023	CoS-MoS_2_	4-Methylphenol	Toluene	1:24	n.r.	180	4	7	Dodecane	85	99.5	[71]
2022	Pt-MoS_2_-x	*p*-Cresol	Methylcyclohexane	1:12	1.6	120	5	10	Dodecane	100	96.3	[72]
2026	Co_x_S_y_-*n*-MoS_2_	*p*-Cresol	Toluene	1:2	<2	170	3	10	Dodecane	99.9	96.7	[73]
2022	Mo-Co_9_S_8_/Al_2_O_3_	Diphenyl ether	Benzene	1:4	n.r.	300	4	4	Methylcyclohexane	98.9	87.2	[74]
2025	Mo/Ti	Phenol	Benzene	N/A	26	330	0.1	1	None (gas phase)	22	100	[75]
2022	Mo_2_C-PC	Guaiacol	Benzene	N/A	7.4	350	0.1	2.5	Decalin	100	70	[76]
2023	Mo_2_N@NC500 /SBA-15	Guaiacol	Aromatic compounds	N/A	2–3	380	2	3	Decane	99	92	[77]

n.r.: not reported; N/A: not applicable.

**Figure 6:**
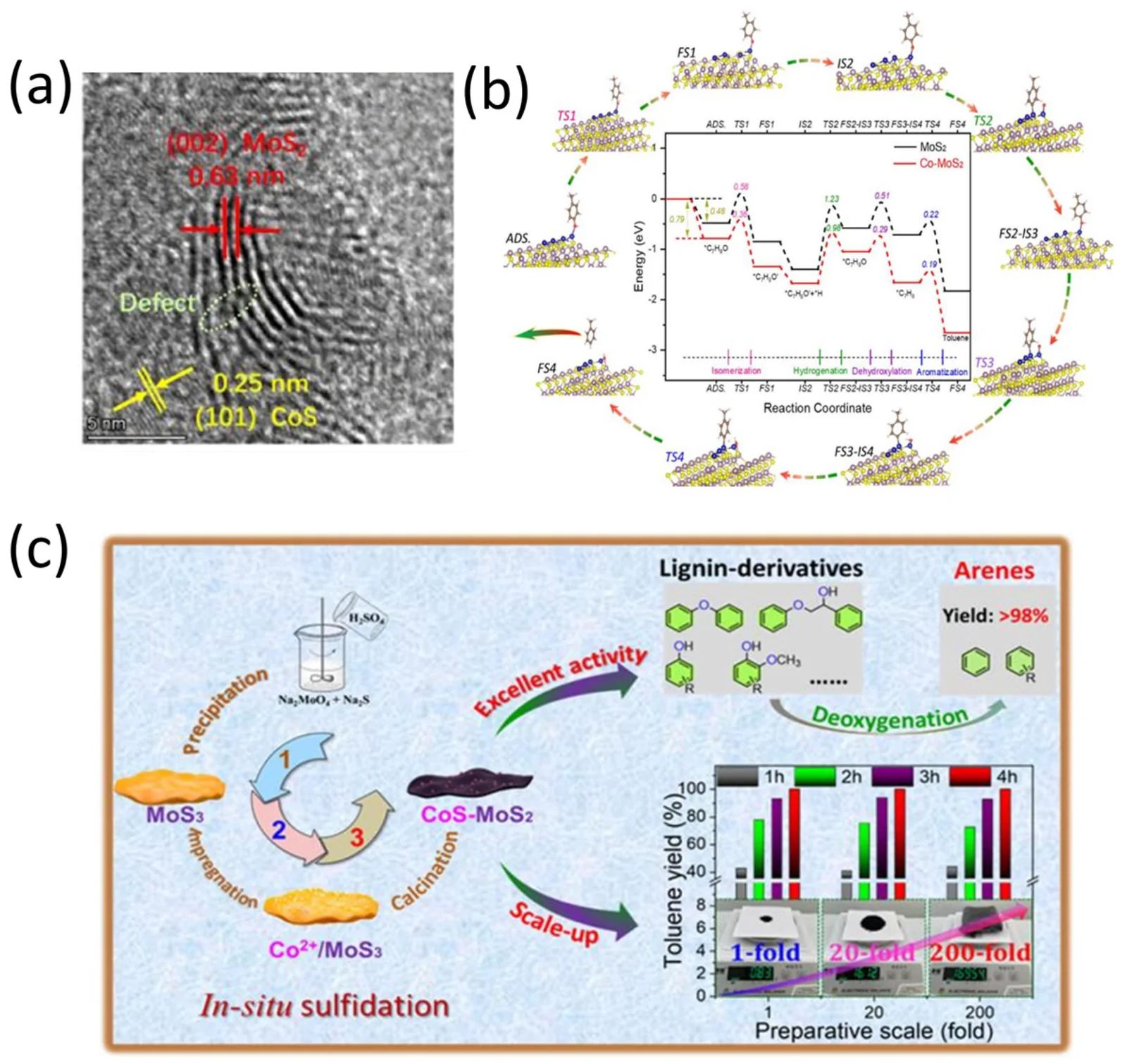
(**a**) HRTEM image of Co-Mo-0.5-400. (**b**) Calculated energy profile for the direct deoxygenation of 4-methylphenol on MoS_2_ and CoS-MoS_2_ catalysts and DFT-optimized structure for the initial state (IS), transition state (TS), and final state (FS) on CoS-MoS_2_ catalyst. (**c**) Flowchart of the preparation process and overview of the properties of CoS-MoS_2_ catalysts. Reproduced with permission from [71]; published by American Chemical Society, 2023.

Among the various strategies, the introduction of promoters at the MoS_2_ edges to construct metal–sulfide interfaces has proven to be an effective approach for enhancing the intrinsic activity of the edge sites. Wang et al. [71] developed a novel strategy for the scalable fabrication of Co-promoted MoS_2_ catalysts by exploiting sulfur vacancies in MoS_3_ and their preferable adsorption of Co ions. Specifically, the CoS-MoS_2_ catalyst was prepared via decomposition of MoS_3_ followed by in situ sulfidation of cobalt oxides. HRTEM observations revealed that CoS tended to cover the edges of MoS_2_, facilitating the formation of CoS–edge interfaces. Compared with pristine MoS_2_, the MoS_2_ fringes in the Co-Mo-0.5-400 catalyst exhibited increased discontinuity and curvature, indicating basal plane rupture and the generation of additional edge sites. This suggested that the addition of Co reduced the catalyst particle size and produced more intrinsic edges, primarily due to the inhibitory effect of Co on MoS_2_ crystallite growth (Figure 6a). Density functional theory (DFT) calculations (Figure 6b) demonstrated that the formation of CoS–edge interfaces enhanced the adsorption of 4-methylphenol and lowered all reaction energy barriers compared to pristine MoS_2_, enabling a toluene yield as high as 100% at a low temperature of 120 °C (Figure 6c). The catalysts synthesized based on this principle exhibited excellent stability, with negligible changes in 4-methylphenol conversion and toluene selectivity over eight consecutive runs. XRD and HRTEM analyses confirmed that no significant agglomeration or sulfur loss occurred. This approach opens up a new scalable strategy for the fabrication of iron family-element-promoted MoS_2_-based catalysts, provides an effective route for lignin oil upgrading, and demonstrates promising potential for applications in industrial catalysis and biomass conversion.

**Figure 7:**
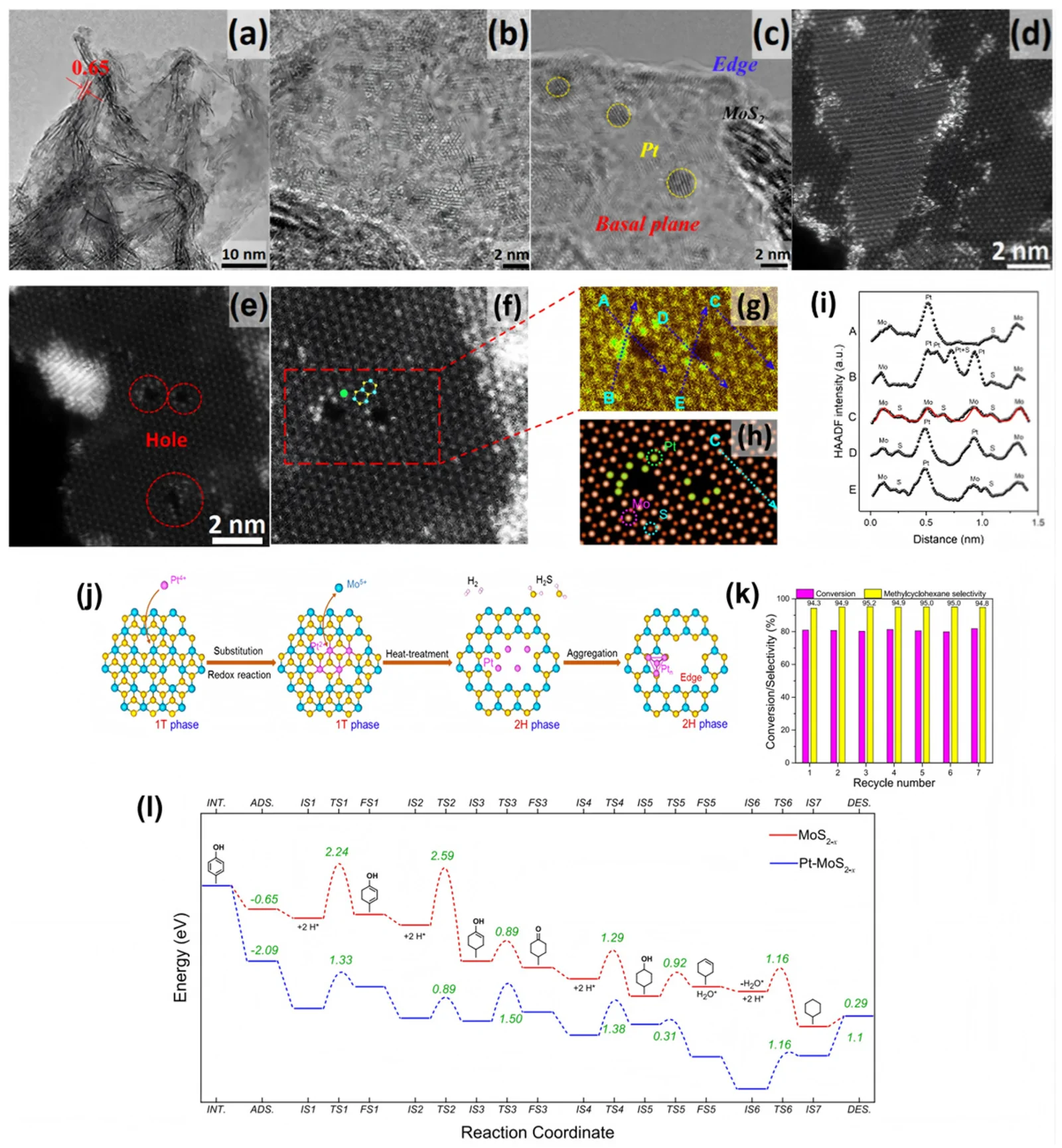
(**a**) TEM image of Pt-Mo. HRTEM images of (**b**) Pt-Mo and (**c**) Pt-Mo-200. HAADF-STEM image of (**d**) Pt-Mo and (**e**) Pt-Mo-200. (**f**,**g**) enlarged and (**h**) simulated HAADF-STEM images of Pt-Mo-200. (**i**) Measured intensity profile along four lines labeled in (**g**), (**h**), and (**j**) schematic illustration of the formation of the Pt-MoS_2_-x catalyst. (**k**) Stability test of Pt-Mo-200 in the HDO of *p*-cresol. (**l**) Energy barrier profiles for the HDO of *p*-cresol on MoS_2_-x and Pt-MoS_2_-x. Reproduced with permission from [72]; published by American Chemical Society, 2022.

As mentioned above, two-dimensional MoS_2_ catalysts exploit edge sites for their catalytic activity, while the basal planes remain inert in heterogeneous catalysis. To activate these inert basal planes, Wu et al. [72] developed a metal intercalation–deintercalation process to prepare a Pt-MoS_2_-x catalyst. HAADF-STEM images revealed that Pt^4+^ partially substituted Mo^4+^ in the basal planes of 1T-MoS_2_-x; upon reduction, Pt was deintercalated from the basal planes, ultimately generating abundant edge sites within the originally inert basal planes of MoS_2_ and forming numerous Pt–edge interfaces (Figure 7a–j). This Pt-MoS_2_-x catalyst exhibited high activity in the HDO of *p*-cresol, achieving 100% oxygen removal at 120 °C, with negligible sulfur loss or deactivation over recycling runs (Figure 7k). DFT calculations indicated that the hydrogenation of the aromatic ring and conjugated diene exhibited the highest energy barriers on MoS_2_-x, identifying these two hydrogenation steps as the primary rate-determining steps. However, owing to the formation of Pt–edge interfaces, dissociated H species on Pt clusters could rapidly spill over to sulfur vacancies at the edge sites to participate in hydrogenation reactions. Consequently, the energy barriers for the aforementioned two rate-limiting hydrogenation steps on the Pt-MoS_2_-x catalyst were significantly reduced, correlating well with the observed catalytic performance and highlighting the critical role of Pt–edge interface formation (Figure 7l). The Pt-MoS_2_-x catalyst was also effective for the HDO of various lignin model compounds and lignin-derived bio-oils. This approach opens up an innovative strategy for designing durable MoS_2_ catalysts with activated basal planes and provides an efficient alternative for the synthesis of lignin-based jet-fuel-range cycloalkanes.

**Figure 8:**
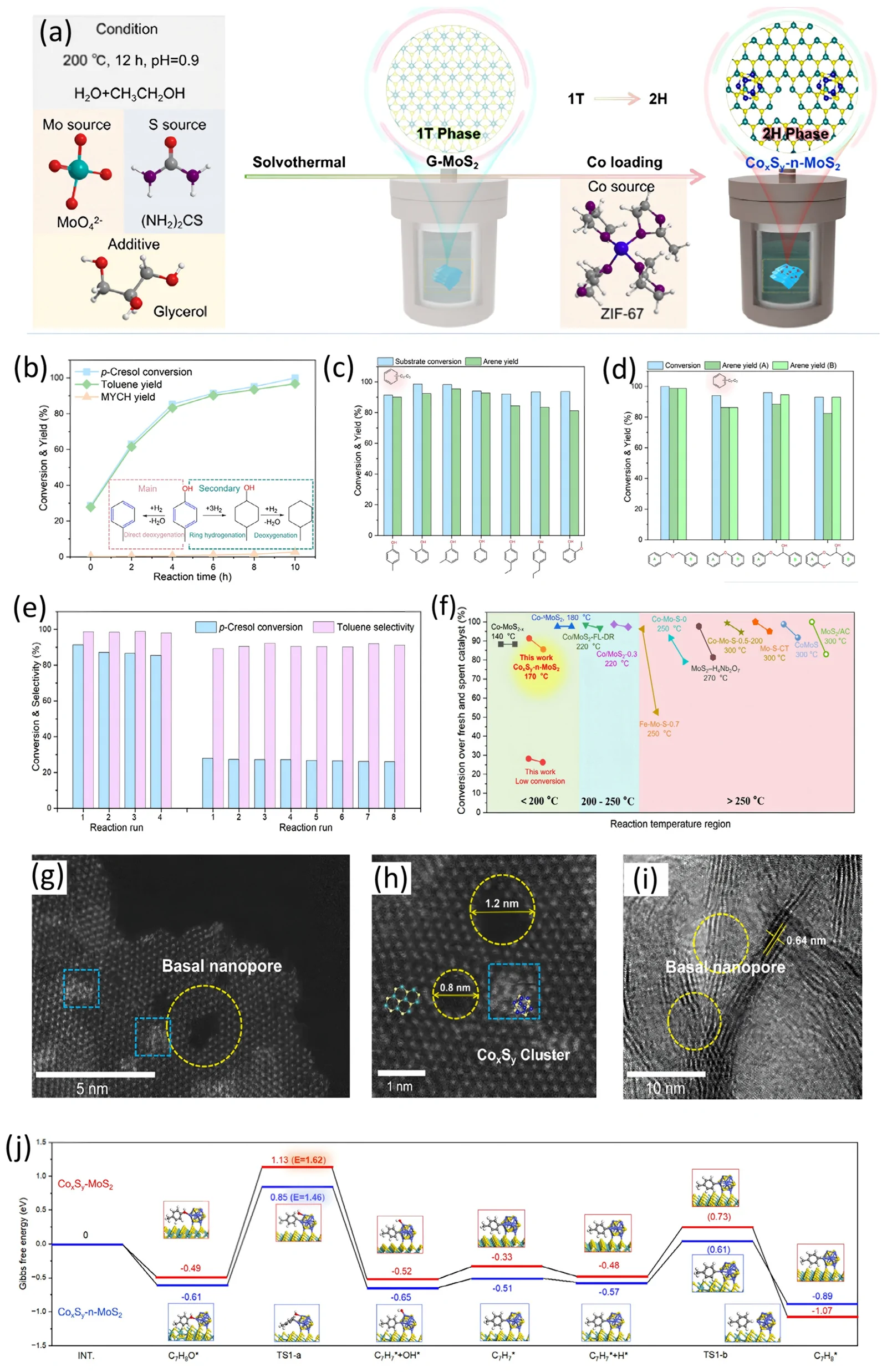
(**a**) Schematic illustration of the preparation procedure of Co*_x_*S*_y_*-*n*-MoS_2_. (**b**) Product distributions as a function of reaction time for Co*_x_*S*_y_*-*n*-MoS_2_. (**c**) HDO result of *p*-cresol over Co*_x_*S*_y_*-*n*-MoS_2_ at different reaction temperatures. (**d**) HDO result of *p*-cresol over Co*_x_*S*_y_*-*n*-MoS_2_ under different initial H_2_ pressure. (**e**) Stability test of Co*_x_*S*_y_*-*n*-MoS_2_ in the HDO of *p*-cresol at 170 °C for 6 h under 3 MPa (full conversion) and 1 MPa (low conversion), respectively. (**f**) The comparison of the activity over various MoS_2_-based catalysts in HDO of lignin derivatives. (**g**,**h**) HAADF-STEM images of Co*_x_*S*_y_*-*n*-MoS_2_. (**i**) HRTEM images of Co*_x_*S*_y_*-*n*-MoS_2_. (**j**) Energy barrier profiles for the HDO of *p*-cresol on Co*_x_*S*_y_*-MoS_2_ and Co*_x_*S*_y_*-*n*-MoS_2_. The yellow and green represent electron accumulation and depletion, respectively. Reproduced with permission from [73]; published by John Wiley and Sons, 2026.

In addition, Diao et al. [73] proposed an alternative one-step approach for activating the inert basal planes of MoS_2_, which involves creating nanopores within the basal planes and confining ultra-small Co*_x_*S*_y_* clusters in close proximity to the nanopores through adjacent Co-S covalent bonds. In this work, a catalyst composed of Co*_x_*S*_y_* clusters supported on a nanoporous MoS_2_ substrate (designated as Co*_x_*S*_y_*-*n*-MoS_2_) was synthesized via a phase transition of 1T-MoS_2_ and in situ sulfidation of a cobalt dimethylimidazole complex (Figure 8a). This catalyst exhibited excellent performance in the HDO of lignin and its derivatives to aromatic hydrocarbons. In the HDO of *p*-cresol, the catalyst achieved 99.9% conversion and 96.7% toluene selectivity under mild conditions (170 °C, 3 MPa H_2_, 10 h), and maintained its performance over eight consecutive cycles. Moreover, the catalyst demonstrated general applicability to G-type (guaiacyl) and H-type (*p*-hydroxyphenyl) lignin monomers with various side-chain groups, representing state-of-the-art performance among reported catalysts (Figure 8e–i). Mechanistic studies revealed that Co was anchored as Co*_x_*S*_y_* clusters via Co-S covalent bonds adjacent to the nanopores on the MoS_2_ basal planes (Figure 8b–d). The ultra-small Co*_x_*S*_y_* clusters served as the primary active sites, while the nanopores contributed to weakening the C-O bonds through a unique stretching effect on the electron-rich aromatic rings (Figure 8j). This work opens up new opportunities for exploring advanced functional MoS_2_-based catalysts for the synthesis of lignin-derived aromatics.

**Figure 9:**
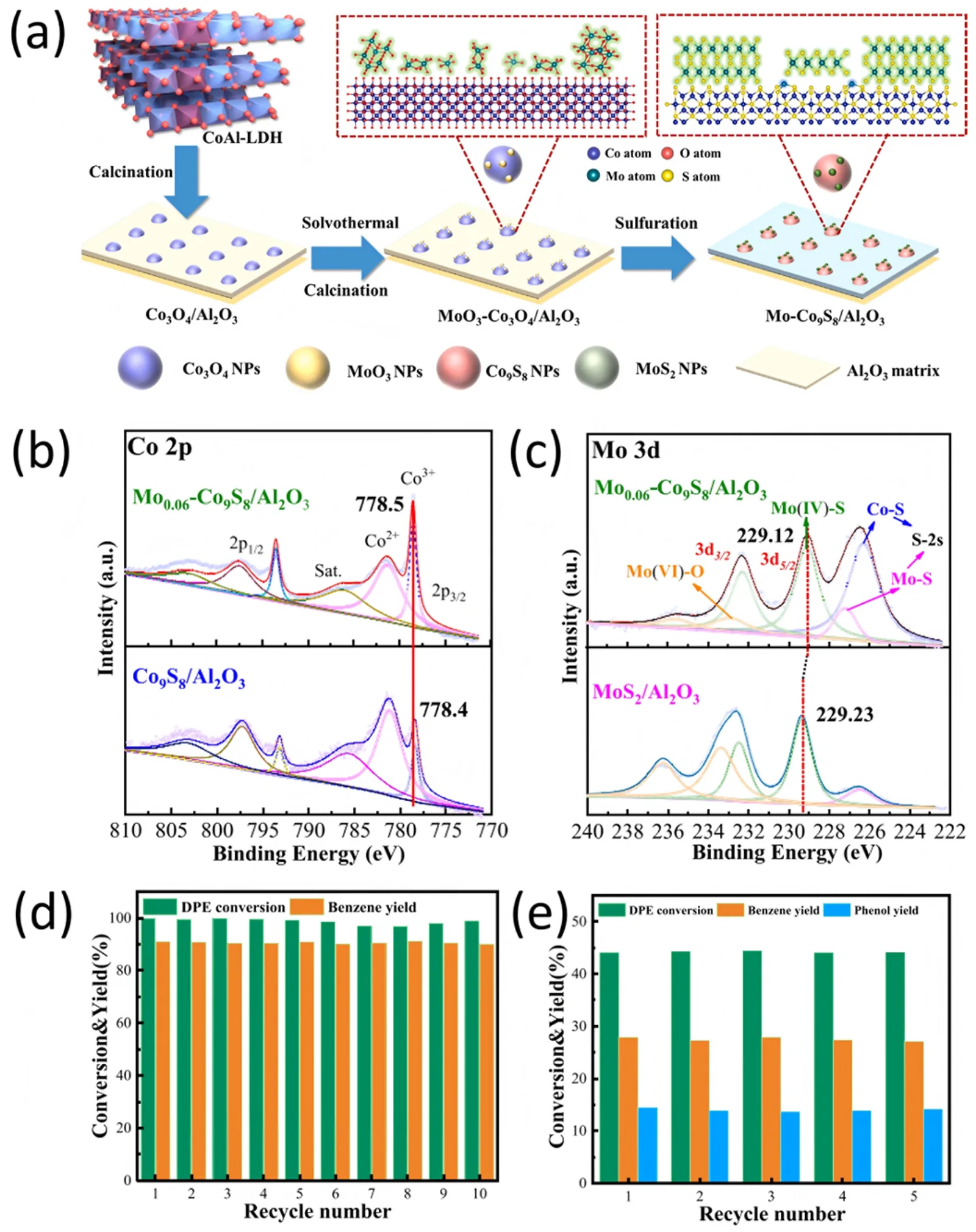
(**a**) Schematic illustration of the preparation procedure of Mo-Co_9_S_8_/Al_2_O_3_ catalyst. (**b**) XPS spectra for Co 2p. (**c**) XPS spectra for Mo 3d. (**d**) Stability test of Mo_0.06_-Co_9_S_8_/Al_2_O_3_ in the HDO of diphenyl ether (DPE) at 265 °C for 10 h. (**e**) Stability test of Mo_0.06_-Co_9_S_8_/Al_2_O_3_ in the HDO of DPE at 265 °C for 2 h. Reproduced with permission from [74]; published by Elsevier, 2022.

In industry, CoMoS and NiMoS are two conventional transition metal sulfide catalysts used in hydrotreating processes, exhibiting good conversion performance (Figure 5b) [82]. However, these sulfide catalysts suffer from sulfur loss and poor stability under industrial operating condition [83,84]. Therefore, to facilitate the practical application of MoS_2_-based catalysts, effective strategies are urgently needed to substantially enhance their high-temperature stability without compromising catalytic activity. Diao et al. [74] developed a facile synthesis strategy to fabricate a Mo-Co_9_S_8_/Al_2_O_3_ catalyst assembled from isolated MoS_2_ slabs and Mo-Co_9_S_8_ structures, which served as a highly selective and stable catalyst for converting lignin model compounds, lignin bio-oil, and even raw lignin into aromatic hydrocarbons. The Mo-Co_9_S_8_/Al_2_O_3_ catalyst was prepared by loading Mo onto a hydrotalcite-derived Co_3_O_4_/Al_2_O_3_ support, followed by subsequent sulfidation. In this catalyst, Mo existed in two forms: isolated MoS_2_ slabs and Mo-Co_9_S_8_ structures, which are chemically connected via Mo-S-Co type covalent bonds on the Co_9_S_8_ surface (Figure 9a). This multifunctional assembled Mo-Co_9_S_8_/Al_2_O_3_ catalyst exhibited electron transfer from Co to Mo sites within the Mo-Co_9_S_8_ structure, and the Mo-Co_9_S_8_ interface possessed a unique affinity for adsorbing oxygenated compounds, thereby conferring exceptional HDO performance (Figure 9b,c). Moreover, owing to the anchoring effect of the Al_2_O_3_ matrix and the electronic effect of Co_9_S_8_, the catalyst demonstrated excellent resistance to high reaction temperatures (265 °C) and H_2_O poisoning. After 10 cycles, the conversion and benzene yield remained at approximately 99.8% and 91.0%, respectively, with no significant deactivation observed (Figure 9d,e). Characterization by XRD, Raman, TEM, and ICP-OES confirmed that both the surface Mo active centers and the overall catalyst structure remained stable after reaction, with no appreciable sulfur loss. Overall, this work provides a facile strategy for designing MoS_2_-based catalysts with exceptional high-temperature stability and HDO activity, highlighting their promising potential for application under harsh high-temperature conditions.

Overall, by virtue of the inherent selectivity toward aromatic hydrocarbons, cost-effectiveness, and sulfur resistance imparted by the edge–rim structure of MoS_2_ itself, together with the morphological tunability arising from its layered structure [85], MoS_2_-based catalysts can achieve efficient and selective deoxygenation of lignin derivatives under mild conditions. However, these systems require high hydrogen pressures and the continuous addition of sulfur to maintain activity, which in turn necessitates additional purification steps to eliminate sulfur contaminants [86]. Moreover, owing to the high oxygen-to-sulfur ratio in lignin oil, sulfide catalysts are susceptible to deactivation through oxidation of the active sites [87]. In contrast, oxides, carbides, and nitrides exhibit activities comparable to those of sulfides and have therefore attracted considerable attention for their application in HDO reactions.

Studies have revealed that molybdenum oxides can selectively cleave C-O bonds under low hydrogen pressures while avoiding saturation of the aromatic rings in lignin-derived compounds, and they also exhibit strong resistance to water and coke formation [88,89,90,91,92,93]. Molybdenum oxides exist in multiple oxidation states, such as MoO_2_ and MoO_3_, and the activity of molybdenum-based oxides is closely related to the electron density of Mo in the catalyst. A comparative study on the HDO performance of various unsupported molybdenum-based compounds toward 4-methylphenol revealed that the turnover frequency (TOF), based on CO uptake, decreased in the order MoP > MoS_2_ > MoO_2_ > MoO_3_, whereas the activation energy increased in the reverse order [94]. Several studies have demonstrated the HDO activity of MoO_2_/C catalysts and their excellent selectivity toward C-O bond cleavage without causing saturation of C=C bonds, thereby reducing hydrogen consumption during the reaction. Ansaloni et al. [88] conducted mechanistic investigations on molybdenum oxides and attributed the HDO performance of MoO_2_/C toward guaiacol to active sites formed by oxygen vacancies (also referred to as coordinatively unsaturated sites, CUS), which simultaneously served as selectivity-determining sites. The surface modifications induced by the formation of CUS influenced both selectivity and activity, and these oxygen vacancies were generated by the surface reduction of specific Mo (IV) species. Furthermore, MoO_2_ exhibits superior resistance to CO formation and water poisoning [88,92,93].

Bulk molybdenum oxides are highly effective for the HDO of various biomass-derived compounds, primarily yielding aromatic hydrocarbons and olefins [95]. The influence of supports on the HDO performance of molybdenum oxide catalysts for lignin-derived compounds has also been subject to preliminary investigation. Studies have indicated that MoO*_x_* catalysts supported on SiO_2_, γ-Al_2_O_3_, TiO_2_, ZrO_2_, and CeO_2_ exhibit exceptionally high selectivity for cleaving the C-O bond of m-cresol without hydrogenating the aromatic ring [91]. In addition, Dionizio et al. [75] conducted a comparative study on the HDO of phenol over molybdenum oxides supported on titania, activated carbon, and silica. The catalytic activity decreased in the order Mo/Ti > Mo/C > Mo/Si, whereas the selectivity to benzene remained 100% across all catalysts, indicating that the molybdenum active phase did not directly influence the reaction selectivity and that the reaction proceeded predominantly via the direct deoxygenation pathway rather than the aromatic ring hydrogenation route. Hydrogen pretreatment exerted a significant effect on catalyst activity: for Mo/Ti, high-temperature reduction (≥400 °C) led to decreased activity, which may be attributed to the SMSI effect; for Mo/C, however, high-temperature reduction (≥400 °C) substantially enhanced the activity, possibly due to the formation of MoO_2_ active sites. In conjunction with H_2_O-TPD results, the amount of hydrogen desorbed followed the order Mo/Ti > Mo/C > Mo/Si and showed a positive correlation with the HDO rate, suggesting that a higher concentration of oxygen vacancies was associated with higher HDO activity. This finding is consistent with the previously discussed influence of oxygen vacancies on the HDO of lignin-derived substrates.

**Figure 10:**
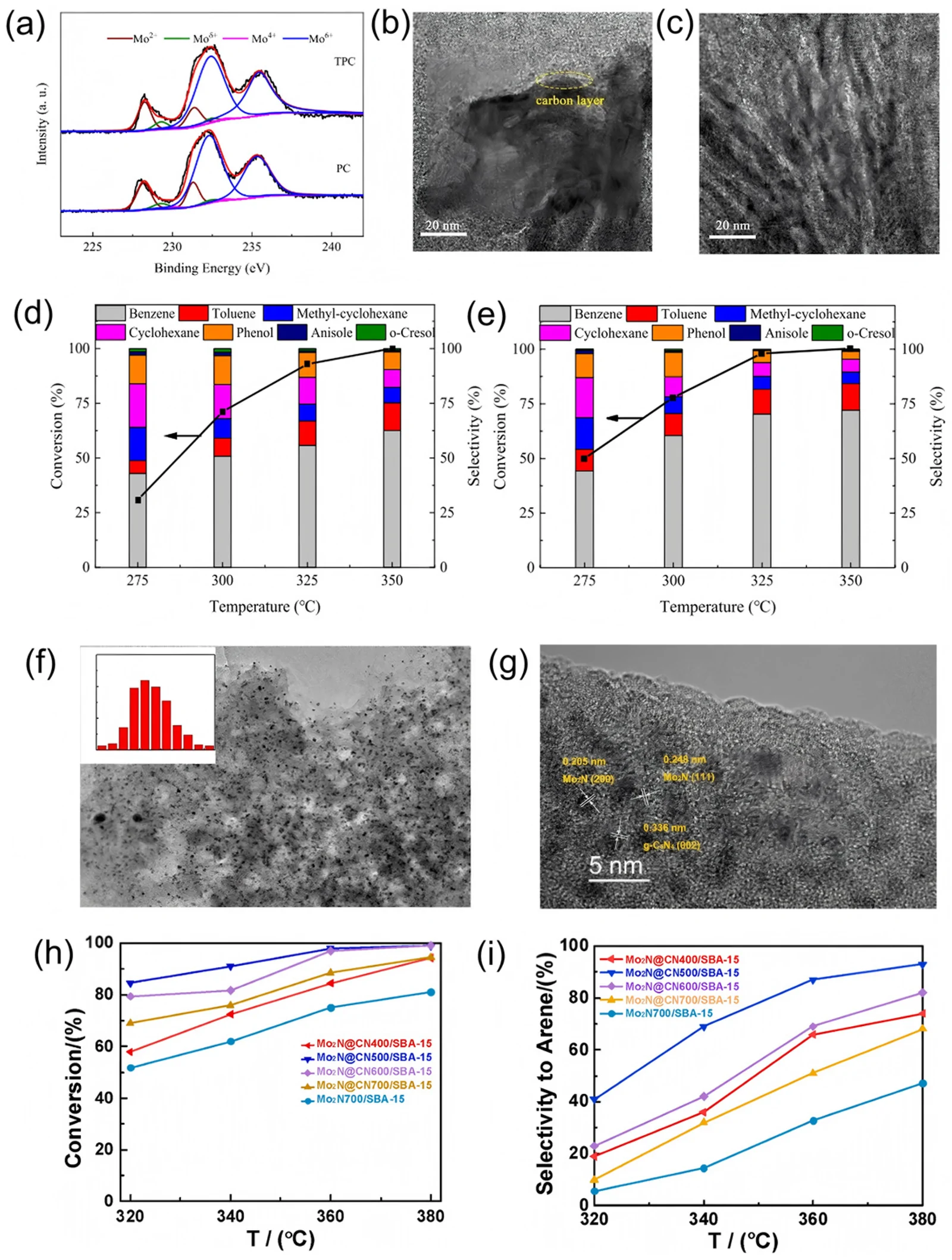
(**a**) Deconvolution of Mo 3d XPS spectra for Mo_2_C-TPC and Mo_2_C-PC. (**b**,**c**) TEM images of Mo_2_C-TPC. HDO of guaiacol over (**d**) Mo_2_C-TPC and (**e**) Mo_2_C-PC. Reproduced with permission from [76]; published by Springer Nature, 2022. (**f**) TEM images of Mo_2_N@CN500 after removing SBA-15. (**g**) HRTEM images of Mo_2_N@CN500/SBA-15 catalyst. Conversion of guaiacol (**h**) and selectivity to arene (**i**) on different Mo_2_N catalyst. Reproduced with permission from [77]; published by Elsevier, 2023.

Recently, molybdenum carbide (Mo_2_C) catalysts have attracted considerable attention due to their Pt-like behavior in HDO reactions [96]. Mo_2_C catalysts have been applied not only to the production of aromatic hydrocarbons [97] but also to the generation of phenolic monomers [98]. However, conventional carburization synthesis methods often lead to carbon deposition on the Mo_2_C surface [99]. To address this issue, Yu et al. [76] employed low-temperature plasma generated from H_2_ and CH_4_ to reduce and carburize molybdenum oxide precursors, yielding β-Mo_2_C, designated as Mo_2_C-PC. For comparison, a molybdenum carbide catalyst (Mo_2_C-TPC) was also prepared via conventional temperature-programmed reduction carburization. According to calculations based on the Scherrer equation from XRD patterns and XPS measurements, Mo_2_C-PC exhibited a smaller crystallite size (7.4 nm) and a higher density of active sites (Mo^2+^) (Figure 10a). TEM characterization revealed that Mo_2_C-PC particles showed no significant agglomeration, whereas aggregated particles and carbon layers were observed in Mo_2_C-TPC (Figure 10b,c). The HDO performance of Mo_2_C-PC and Mo_2_C-TPC was evaluated using guaiacol, phenol, and anisole as substrates in a stainless-steel tubular reactor. The results demonstrated that at low temperatures (<300 °C), Mo_2_C-PC exhibited higher conversion of phenolic compounds and higher selectivity to aromatics compared to Mo_2_C-TPC (Figure 10d,e). In the HDO of guaiacol, the TOF of Mo_2_C-PC (0.37 s^−1^) was comparable to that of Mo_2_C-TPC (0.32 s^−1^), indicating that the intrinsic reaction rate per active site did not differ significantly between the two catalysts. This suggested that the enhanced performance could be attributed to the reduced particle size, increased Mo sites (Mo^2+^), and suppressed carbon deposition. This work provides new insights into the controlled synthesis of molybdenum carbide catalysts and opens up a viable pathway for the low-temperature and high-efficiency production of lignin-based aromatic hydrocarbons.

Mo_2_N exhibits catalytic properties similar to those of platinum-group metals, a feature attributed to the incorporation of nitrogen atoms into the interstitial sites of the molybdenum lattice. Through hybridization between Mo d-orbitals and N p-orbitals, the d-band center of Mo is downshifted, thereby weakening the excessive adsorption of reaction intermediates on the surface and achieving optimized adsorption energetics [100,101]. Consequently, Mo_2_N is also considered a promising catalyst for the HDO of bio-oil [102,103,104,105,106]. Wang et al. [77] employed MoCl_5_ and dicyandiamide as the Mo source and C/N source, respectively, to prepare Mo_2_N@NC/SBA-15 with high stability and dispersity. Mo_2_N was formed in situ during high-temperature carburization under H_2_, while dicyandiamide was converted into nitrogen-doped carbon (NC) (Figure 10f,g). The formation of ultra-fine Mo_2_N nanoparticles (NPs) was attributed to the N-doped carbon species surrounding the Mo_2_N NPs, which prevented their aggregation at the high carburization temperature (Figure 10e). The Mo_2_N@NC/SBA-15 catalyst exhibited high HDO activity and selectivity toward aromatic compounds (Figure 10h,i). At 380 °C and 2 MPa, the selectivity to aromatic products reached 92%, with a guaiacol conversion of 99%, fully demonstrating the great potential of molybdenum-based nitrides for the HDO of lignin to aromatic hydrocarbons.

Across the Mo-based family, the deliberate engineering of undercoordinated Mo sites—edge sites, basal-plane defects, and oxygen vacancies—together with tuning of the Mo oxidation state and support interaction, emerges as the recurring design strategy. These sites govern the balance between C-O cleavage and aromatic-ring hydrogenation: edge-site density and oxygen vacancies favor direct deoxygenation, whereas excessive reduction or sulfur loss can alter selectivity and stability [66,67,68,69,70,71,72]. Therefore, molybdenum-based sulfides exhibit excellent sulfur resistance and HDO performance, rendering them promising for the processing of industrial lignin. However, their practical application is constrained by a series of issues arising from S-O exchange, including sulfur loss, product contamination, water poisoning, poor high-temperature stability, and insufficient activity. Meanwhile, oxides, carbides, and nitrides also suffer from certain drawbacks, including complex preparation procedures and deactivation via oxidation induced by water formation during the HDO process [95,107]. Furthermore, current research has largely focused on the conversion of lignin-derived monomers and model compounds. Although excellent conversion rates and HDO product selectivity have been achieved, the direct application of these catalysts to the HDO of actual lignin for the production of aromatic and cycloalkane fuels holds greater promise for industrial applications and warrants further development [108].

### 2.2. Nickel-Based Catalysts

Nickel-based catalysts have been widely employed in the HDO of lignin derivatives owing to their cost-effectiveness and strong hydrogen dissociation capability. However, in most cases, these catalysts require harsh reaction conditions (e.g., 250 °C, 5 MPa) to convert lignin monomers and C-O bond dimers into cycloalkanes through complete deoxygenation and aromatic ring hydrogenation [109]; their activity under mild conditions remains insufficient and requires further enhancement. In addition, the recalcitrant C-C bonds inherent in the lignin structure are generally difficult to cleave using non-noble metal catalysts, which restricts the product yield from the HDO of raw lignin [29]. Furthermore, nickel-based catalysts are reported to be susceptible to oxidation and agglomeration during application, resulting in decreased stability, which limits their practical utility to some extent. Therefore, enhancing the C-O and C-C bond cleavage capability along with the cycling stability of nickel-based catalysts constitutes a critical challenge that must be addressed [110,111]. Table 2 summarizes recent studies on the application of nickel-based catalysts in the catalytic HDO of lignin and its derivatives.

To address this issue, Diao et al. [112] developed a Ni@AlPO_4_/Al_2_O_3_ catalyst, in which the surface of Ni nanoparticles was modified with AlPO_4_ species. This catalyst exhibited excellent catalytic activity and stability in both C-C and C-O bond cleavage. Under conditions of 230 °C, 1 MPa H_2_, and 2 h, it achieved a guaiacol conversion of 99.1% and a cyclohexane yield of 92.9%. Half-conversion experiments demonstrated that the catalyst maintained good performance after five cycles, showing its robust recyclability. Mechanistic studies revealed that the formation of Ni^δ+^-AlPO_4_ interfaces and the moderate surface coverage of Ni by AlPO_4_ significantly promoted the conversion of methoxycyclohexanol and cyclohexanol to cyclohexane. Notably, the AlPO_4_ coverage exhibited an “optimal window”, that is, insufficient coverage led to inadequate Ni^δ+^-AlPO_4_ interfaces, whereas excessive coverage blocked Ni sites, resulting in a sharp decline in cyclohexane yield. Furthermore, Ni@AlPO_4_/Al_2_O_3_ demonstrated broad substrate applicability in the HDO of various typical lignin monomers and dimers to cycloalkanes. This work provides a successful example of a highly stable and active nickel-based catalyst for converting lignin derivatives into cycloalkanes and lays a foundation for the development of lignin-based sustainable aviation fuel.

**Figure 11:**
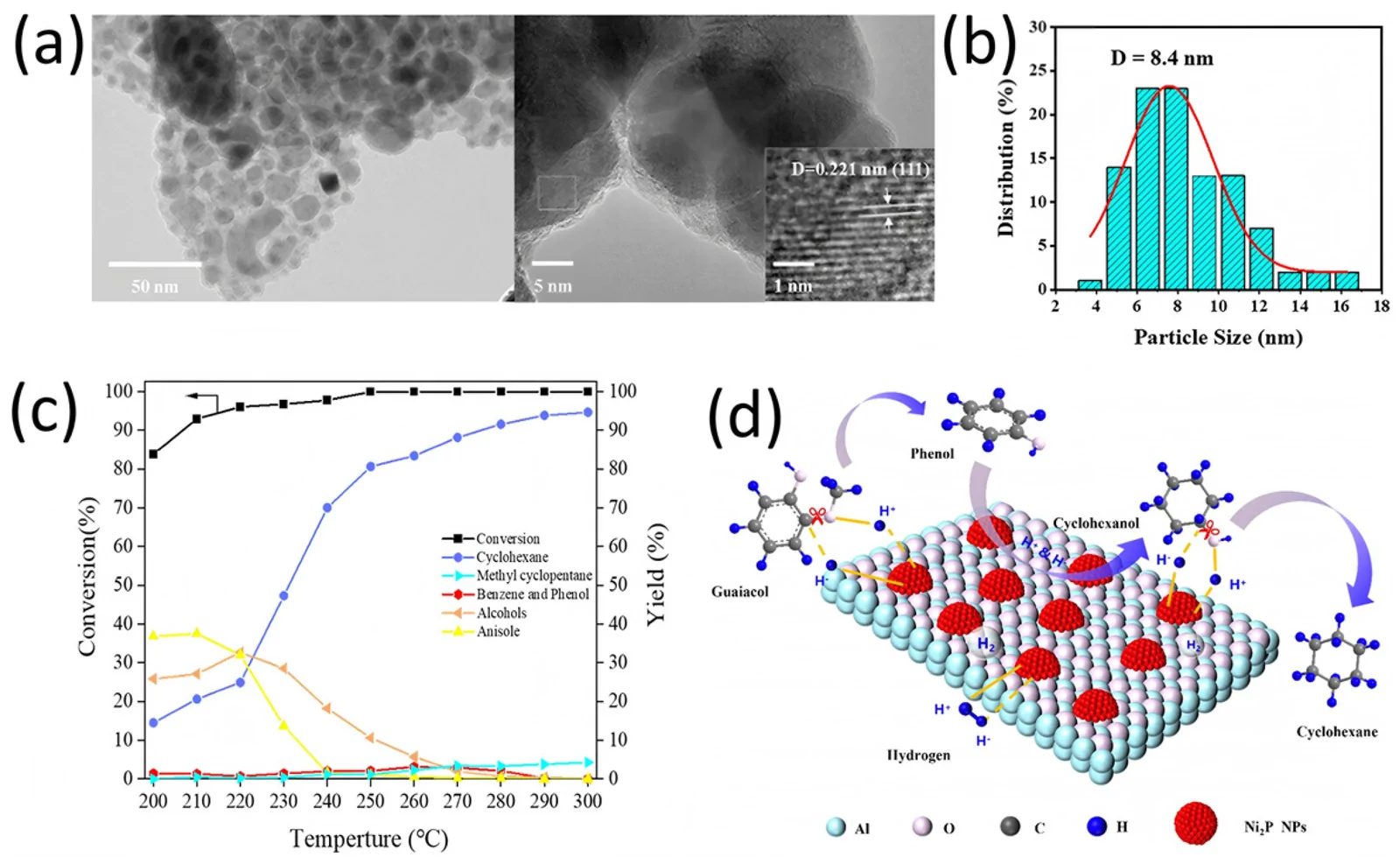
(**a**) TEM images and (**b**) the size distribution of Ni_2_P-Al_2_O_3_(H)71. (**c**) Hydrogenation and deoxygenation performance of Ni_2_P-Al_2_O_3_(H)71. (**d**) Proposed mechanisms for cleavage of C-O bonds and hydrogenation in guaiacol. Reproduced with permission from [113]; published by American Chemical Society, 2022.

Numerous studies have demonstrated that nickel phosphide (Ni_2_P) exhibits superior HDO performance compared to other transition metal phosphide catalysts, a property attributed to the higher surface metal site density and electron density in Ni_2_P [115,116,117]. Ni_2_P contains two distinct types of Ni sites, designated Ni(1) and Ni(2) [118], among which the pyramidal Ni(2) sites preferentially favor the hydrogenation pathway [119]. In general, Ni_2_P catalysts can achieve high alkane yields in the HDO of oxygenated lignin model compounds at relatively elevated temperatures [115]; however, the cyclohexane yield drops sharply when the reaction temperature falls below 300 °C [120]. Studies have revealed that reducing the Ni_2_P particle size while simultaneously enhancing the dispersion of the active phase plays a crucial role in improving the low-temperature catalytic performance [121]. Jia et al. [113] developed a strategy for the low-temperature synthesis of a hydrotalcite-derived nickel phosphide catalyst with a flower-like porous structure (Ni_2_P-Al_2_O_3_). Benefiting from the high dispersibility of hydrotalcite, the resulting Ni_2_P catalyst, obtained after in situ reduction with red phosphorus at 500 °C, exhibited small particle sizes (Figure 11a,b) and abundant active and acidic sites, which facilitated the activation of both substrates and H_2_. Under conditions of 5 MPa H_2_ and 250 °C for 3 h, Ni_2_P-Al_2_O_3_ achieved a guaiacol conversion of 100% and a cyclohexane yield of 94.5% (Figure 11c). Additionally, Ni_2_P-Al_2_O_3_ also displayed high HDO activity in the conversion of lignin oil and poplar lignin. DFT calculations further revealed that, compared to Ni(1) sites, the highly exposed Ni(2) sites on the Ni_2_P-Al_2_O_3_ surface possess a stronger capability for C-O bonds cleavage during the HDO of guaiacol (Figure 11d). This work demonstrates that through nanoscale regulation of particle size and dispersion, Ni_2_P-based catalysts can achieve efficient and selective conversion of lignin derivatives into jet-fuel-range cycloalkanes under mild conditions.

**Figure 12:**
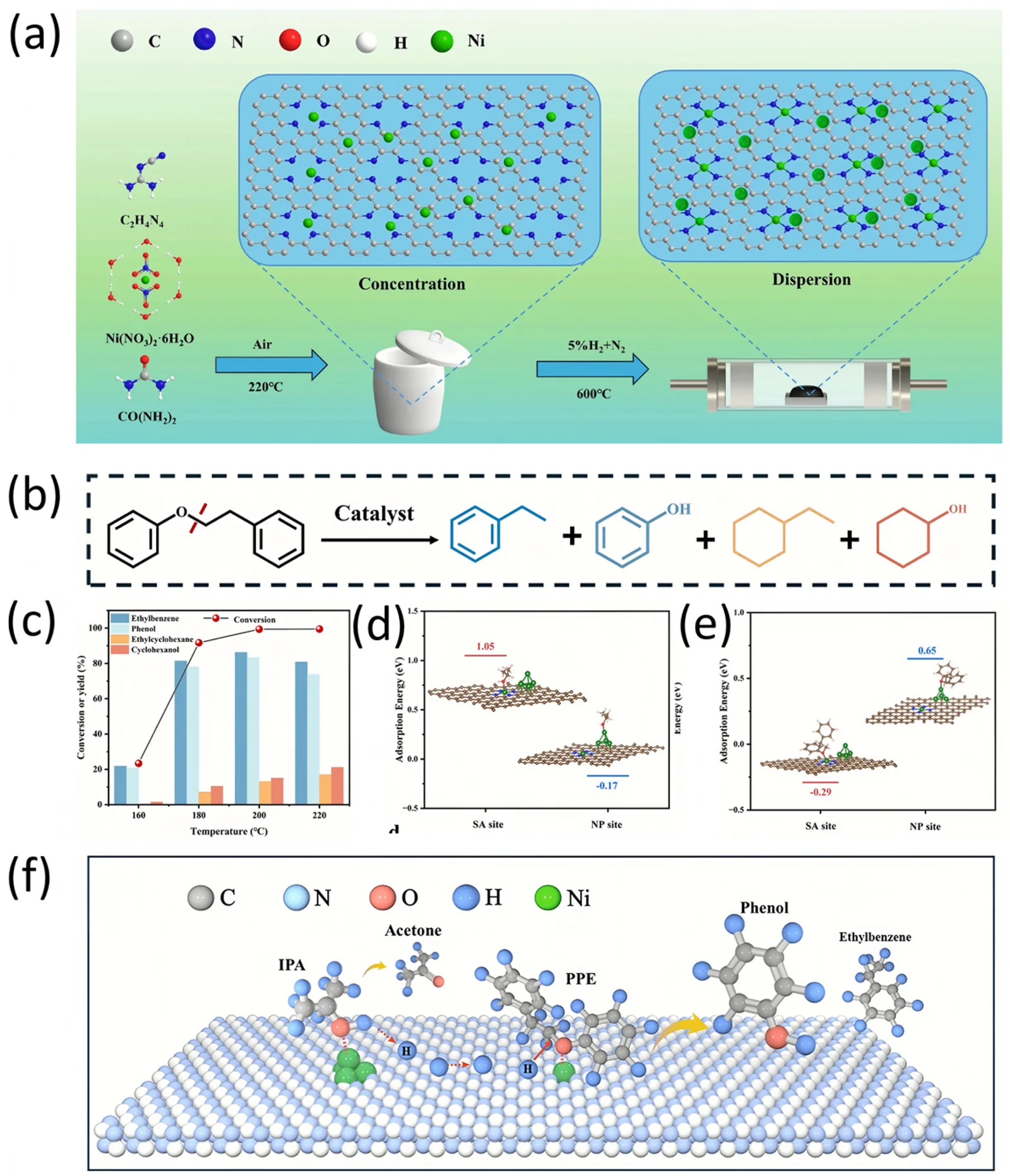
(**a**) Schematic diagram of the preparation method of NiNPs-SAs/NC catalyst. (**b**) Schematic of PPE hydrogenolysis reaction. (**c**) The effect of reaction temperature on hydrogenation deoxygenation of PPE. (**d**) Adsorption energy of isopropanol on different sites of the NiNPs-SAs/NC model. (**e**) Adsorption energy of PPE on different sites of the NiNPs-SAs/NC model. (**f**) Relative energy plots of PPE hydrogenolysis on NiNPs-SAs/NC and NiNPs/NC. Reproduced with permission from [114]; published by Elsevier, 2026.

Conventional HDO of lignin is typically conducted under relatively high hydrogen pressures (>2 MPa) [122,123], which poses significant safety and engineering challenges. To circumvent these issues, catalytic transfer hydrogenation (CTH) has been proposed as an alternative approach, wherein hydrogen atoms are supplied by sacrificial hydrogen-donor solvents (such as methanol, ethanol, and isopropanol) instead of gaseous H_2_, enabling lignin hydrogenolysis under mild or atmospheric pressure [124,125]. Zeng et al. [114] prepared a NiNPs-SAs/NC catalyst, comprising Ni single atoms (SAs) and Ni nanoparticles (NPs) embedded in nitrogen-doped carbon, through an adsorption–aggregation and dispersion–capture strategy (Figure 12a). This catalyst was capable of cleaving C-O bonds in lignin under mild conditions (200 °C, with isopropanol as the hydrogen donor) while suppressing over-hydrogenation of the resulting aromatic monomers, thus affording high selectivity toward aromatic compounds (Figure 12b,c). Furthermore, DFT calculations (Figure 12d–f) were employed to elucidate the catalytic mechanism, revealing a strong synergistic interaction between Ni SAs and Ni NPs within the NiNPs-SAs/NC catalyst. Specifically, isopropanol preferentially adsorbed and dissociated on Ni NP sites to generate H* species, while the C-O bonds of the lignin model compound (PPE) were activated on Ni SA sites. Subsequently, H* species could spill over from Ni NPs to Ni SAs to complete the HDO reaction. This work provides a catalyst capable of selectively cleaving C-O bonds under mild conditions and offers a new strategy for the production of jet-fuel-range aromatics from lignin.

Nickel-based catalysts face a recurring tension: strong hydrogen activation is necessary for C-O cleavage, yet excessive hydrogenation saturates aromatic rings. Size control, site geometry, and spatial separation of functions are the most effective ways to reconcile these competing demands. Although non-noble metals such as Ni are abundant and cost-effective, their synthesis routes are generally more complex, leading to increased process costs. Moreover, most transition metals are typically extracted from sulfides and arsenides, processes that may incur additional environmental costs. Furthermore, unsupported Ni active sites are prone to deactivation via coke deposition and agglomeration in the absence of strong metal–support interactions [126]. In addition, the efficiency of the catalytic conversion of macromolecular lignin over unsupported Ni catalysts is highly dependent on the solvent design and mass-transfer conditions of the reaction system [127]. Consequently, the introduction of a second metal with distinct hydrotreating or oxophilic properties to form bimetallic catalysts, or the incorporation of supports bearing tunable acid sites or redox sites to construct bifunctional catalysts, serves both to modify the active sites of the primary metal and to simultaneously generate additional active sites [46].

### 2.3. Bimetallic Catalysts

Compared with monometallic catalysts, bimetallic catalysts represent an intriguing strategy for HDO, as they offer the possibility to tune catalytic performance and tailor product selectivity. Bimetallic catalyst systems formed by Co, Ni, Mo, W, and noble metals in combination, such as PtSn [128], PtRh [64], NiRe [129], PtRe [130], and ZnPd [131], are among the most commonly employed for lignin HDO, and these bimetallic catalysts generally exhibit high selectivity.

For bimetallic catalysts, the activation effects induced by the addition of a second metal can range from a simple promotional role, e.g., assisting in the reduction of the first metal via hydrogen spillover, to more complex metal–metal interactions. These interactions can generally be classified into five distinct phenomena [28]:

(1) Geometric (or ensemble) effects, wherein the addition of the metal activator alters the geometric structure (or ensemble size) of the active sites composed of the catalytically active metal.
(2) Electronic (or ligand) effects, wherein the metal activator modifies the electronic properties of the active sites composed of the catalytically active metal through inter-metal electron transfer (or by functioning as a ligand).
(3) Stabilization effects, wherein the introduction of the metal additive enhances the stability of the catalytically active metal (e.g., by inhibiting sintering or suppressing the formation of carbonaceous deposits on the catalyst surface).
(4) Synergistic effects, wherein both metal species participate in the chemical bonding with reaction intermediates and transition states.
(5) Bifunctional effects, in which each metal provides a distinct function in the reaction mechanism (e.g., one metal catalyzes hydrogenation–dehydrogenation reactions, while the second metal catalyzes C-O hydrogenolysis), which will be detailed in the following section.

In general, geometric and electronic effects influence catalytic activity and selectivity, whereas stabilization effects enhance catalyst stability. Synergistic and bifunctional effects typically increase reaction rates but may also give rise to new reaction pathways. Table 3 summarizes recent studies on the application of bimetallic catalysts in the catalytic HDO of lignin and its derivatives.

Ru-based catalysts have been widely employed in the HDO of lignin and its derivatives owing to their high performance and relatively lower cost compared to other noble metals [135,136,137,138]. Moreover, the addition of a second, less expensive metal to Ru not only reduces the overall catalyst cost but also allows for the modulation of catalytic performance (including selectivity, activity, coke resistance, and durability) through electronic effects [139]. Li et al. [132] applied a bimetallic RuNi/SiO_2_-ZrO_2_ catalyst to the HDO of guaiacol and observed a strong interaction between the two metals, with electron transfer from Ru to Ni. This resulted in a shift in the d-band center of the surface Ni atoms, thereby endowing the catalyst with adsorption properties distinctly different from those of monometallic Ru and Ni, particularly significantly enhanced adsorption of both the substrate and hydrogen, leading to a cyclohexane yield approaching 100%. Furthermore, the bimetallic RuNi catalyst also exhibited good HDO performance toward other phenolic compounds and raw lignin oil. This work highlights the critical role of electronic effects in modulating adsorption properties and HDO activity.

**Figure 13:**
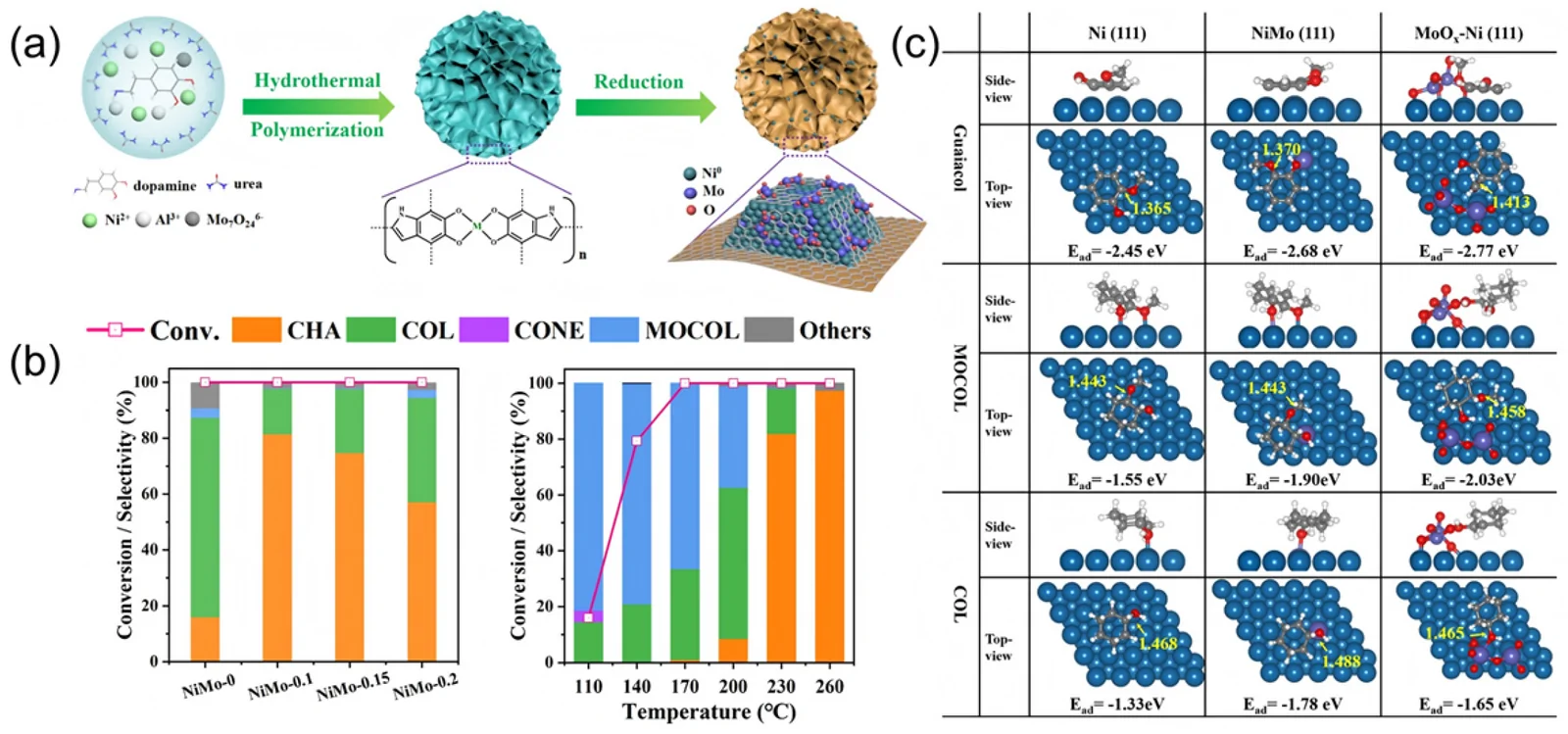
(**a**) Schematic Illustrations of the Synthesis of Surface MoO*_x_*-Decorated Ni-Mo Bimetallic Catalysts via a Facile Dopamine-Assisted Hydrothermal Approach. (**b**) HDO behavior of guaiacol under different catalysts and at different temperatures. (**c**) Optimized geometries for guaiacol, MOCOL, and COL adsorption configurations on Ni (111), Ni-Mo (111), and MoO*_x_*-Ni (111) model surfaces. Reproduced with permission from [133]; published by American Chemical Society, 2021.

The synergistic effect of bimetallic combinations can enable transition metals to exhibit catalytic performance comparable to that of noble metals. Zhang et al. [133] prepared a MoO*_x_*-decorated bimetallic Ni-Mo catalyst with a unique hierarchical flower-like micro/nanoarchitecture via a facile dopamine-assisted hydrothermal method (Figure 13a), and applied it to the HDO of guaiacol for cyclohexane production. Structural characterization revealed that, after high temperature reduction, Ni-Mo alloy sites coexisted with surface-defective MoO*_x_* species on the catalyst surface. By tuning the Mo content, the bimetallic Ni-Mo catalyst with a Mo/Ni molar ratio of 0.1 exhibited superior HDO performance compared to the Mo-free catalyst, as well as to Al_2_O_3_-supported Ni and bimetallic Ni-Mo catalysts prepared by impregnation (Figure 13b). DFT calculations revealed that the surface-defective MoO*_x_* species adjacent to the metal sites promoted the demethoxylation of guaiacol and its reaction intermediates, while the Ni-Mo alloy sites facilitated the dehydroxylation of the cyclohexanol intermediate (Figure 13c). Thus, the cooperative effect between the Ni-Mo alloy sites and the defective MoO*_x_* structures, wherein both metal species participate in the chemical bonding at different steps of the reaction, plays a crucial role in accelerating the demethoxylation and dehydroxylation processes during guaiacol HDO. This surface-defect bimetallic engineering approach provides promising guidance for the construction of highly efficient bimetallic catalysts for the production of lignin-based cycloalkanes.

Selective conversion of lignin to cycloalkanes under mild conditions remains a significant challenge [122], as the cleavage of aromatic C-O bonds requires a bond dissociation energy as high as 468 kJ mol^−1^ [111]. Exerting bimetallic interactions offers a promising avenue to overcome this obstacle. Chen et al. [134] developed a novel two-dimensional NiO nanosheet-supported Pd-Pt alloy catalyst (Pd_1.7_-Pt_0.3_/NiO), which enabled efficient HDO of lignin-derived phenolic compounds (conversion > 95%) under near-ambient pressure (1 atm). Mechanistic studies revealed that the catalyst operated via a dual-active-site synergistic cascade mechanism: the Pd-Pt alloy nanoparticles served as the primary active sites for hydrogen activation and substrate conversion, while the NiO support synergistically enhanced aromatic ring hydrogenation through hydrogen spillover. The outstanding catalytic performance was attributed to the synergistic contribution of three factors: firstly, the hydrogen spillover synergy between the alloy sites and the NiO support enabled the relay of aromatic ring hydrogenation and C-O bond cleavage across the two types of sites; secondly, the average particle size of the alloy nanoparticles was only 3.35 nm (substantially smaller than the 5.14 nm of Pd_2_/NiO), and this reduced particle size significantly increased the number of metal–support interfacial sites; and thirdly, the formation of the Pd-Pt alloy modulated the electronic structure of the active metals, enhancing the Lewis acidity at the metal–support interface, thereby promoting the adsorption of the cyclohexanol intermediate and facilitating C-O bond cleavage. This work provides a new catalyst design strategy for the synthesis of lignin-based jet-fuel-range cycloalkanes under mild conditions.

In summary, bimetallic systems provide a versatile platform for tuning deoxygenation selectivity through geometric, electronic, stabilization, and synergistic effects [28]. It should be noted that, despite the evident advantages of using bimetallic catalysts, several challenges remain to be addressed. First, carbon deposition on (Co, Ni)- and (Mo, W)-based catalysts [140] constitutes a major issue in HDO: coking increases with catalyst acidity, yet acid sites are inherently required in the conventional HDO mechanism [141]. Second, the interactions between catalysts and impurities are not yet well understood. This problem becomes particularly prominent when real feedstocks containing various impurities are employed. Consequently, more fundamental studies are needed to establish correlations between the structural features of real lignin feedstocks and the corresponding catalytic performance.

### 2.4. Bifunctional Catalysts

When the second metal does not function as a metal per se, but rather introduces a second type of functionality to the catalyst, a different type of activation effect arises. For example, the presence of metal oxides can introduce acid sites or redox sites. In such cases, the catalyst generally operates through bifunctional synergy, which may alter the reaction selectivity compared to pure metal catalysts. The boundary between bimetallic catalysts and bifunctional catalysts is not always well-defined, because when one of the metals is not fully reduced, metal oxides may form and serve as a support that provides secondary active sites. In such cases, the catalyst should be classified as bifunctional. Whether a catalyst is defined as bimetallic or bifunctional is ultimately determined by the degree of metal reduction [28]. In recent years, bifunctional catalysts have achieved breakthrough progress among numerous catalytic materials owing to their superior performance, and have become a growing research focus [142,143]. Table 4 summarizes recent studies on the application of bifunctional catalysts in the HDO of lignin and its derivatives.

#### 2.4.1. Metal-Zeolite Composite Bifunctional Catalysts

Zeolites have been widely employed in biomass valorization owing to their tunable Brønsted/Lewis acid sites, well-defined pore structures, and shape-selective catalytic capabilities, which render them highly effective in promoting reactions such as cracking, aromatization, and isomerization. In the HDO of lignin derivatives, ZSM-5 stands out for aromatics production due to its moderate pore dimensions and relatively strong acidity; zeolite Y, with its larger pore sizes, facilitates the diffusion and conversion of bulky reactant molecules; and zeolite β, characterized by its three-dimensional intersecting channel systems and medium-strength acidity, also exhibits favorable reaction adaptability. These three zeolite systems are currently the main focuses of research in this field.

However, the practical application of zeolite catalysts alone still faces challenges such as incomplete deoxygenation, unsatisfactory selectivity toward aromatics, and rapid catalyst deactivation due to coke deposition [151]. The HDO of lignin typically involves a tandem process comprising C-O bond cleavage and aromatic ring hydrogenation, which requires the efficient transfer of active hydrogen species [152]. Therefore, by combining metal active sites with the acidic properties of zeolites, the development of metal–zeolite-integrated catalysts enables the construction of a micro-reaction system with a confined adjacency structure at the nanoscale. In this system, the metal component (e.g., Pt, Ni) is primarily responsible for hydrogen activation and selective deoxygenation, while the zeolite governs C-C bond cleavage, isomerization, and aromatization through its acid sites and shape-selective pore channels [153,154]. The synergy between the two functionalities not only optimizes the reaction pathway and lowers the reaction energy barrier, but also enhances the yield and selectivity of target hydrocarbon products such as aromatics, while simultaneously improving coking resistance and extending the catalyst lifetime.

Highly active bifunctional catalysts consisting of metal nanoparticles supported on various zeolites exhibit unique advantages in catalyzing HDO [155]. For inexpensive non-noble metals, particularly nickel-based bifunctional catalysts, their intrinsic atomic structure features a broader d-electron band and higher energy density, which endow them with strong H_2_ activation capability and enhanced catalytic activity under mild conditions [156]. Correspondingly, zeolite supports such as HZSM-5, HY, and Hβ possess three-dimensional channel-type micropores that enhance both thermal and hydrothermal stability, thereby improving the HDO performance of the bifunctional catalysts [157,158]. Furthermore, hydrogenation predominantly occurs on the exposed metal centers, while deoxygenation primarily takes place at the acid sites of the zeolite [158]. Therefore, establishing the relationship between the acid strength and acid amount of zeolite supports and the cleavage of C-O bonds is of great significance for the catalytic HDO of lignin. Lee et al. established a linear correlation between cyclohexane yield and acid amount in Pt/zeolite-HY and Pt/zeolite-Beta catalysts for the HDO of guaiacol [159]. Kang et al. [144] prepared a series of Ni nanoparticle (NP)-loaded HZSM-5 (HZ-5) catalysts with different Si/Al ratios (*n* = 25, 50, 170, and 200) via a modified deposition–precipitation method, and applied them to the HDO of lignin-related model compounds (LRMCs) containing various C-O bonds and aromatic rings, including oxydibenzene (ODB), oxybis(methylene)dibenzene (OBMDB), benzyloxybenzene (BOB), and oxydinaphthalene (ODN). The catalytic HDO mechanisms of ODB, OBMDB, BOB, and ODN over Ni_13%_/HZ-5(n) were subsequently investigated. The results revealed that, with the synergy of Ni NPs, Ni/HZ-5(n) could activate H_2_ to form diatomic hydrogen (H⋯H) and heterolytically split H⋯H into relatively mobile H^+^ and immobile H^−^, further inducing C-O bond cleavage to varying extents via active hydrogen transfer. This mechanism clarifies the different roles of metal sites and acid sites in C-O bond cleavage: metal sites govern hydrogen activation and transfer, whereas acid sites promote the activation and dissociation of C-O bonds. This thereby provides a theoretical basis for the rational design of metal–acid sites in bifunctional catalysts and offers valuable guidance for the HDO of lignin to produce jet-fuel-range cycloalkanes and aromatics.

**Figure 14:**
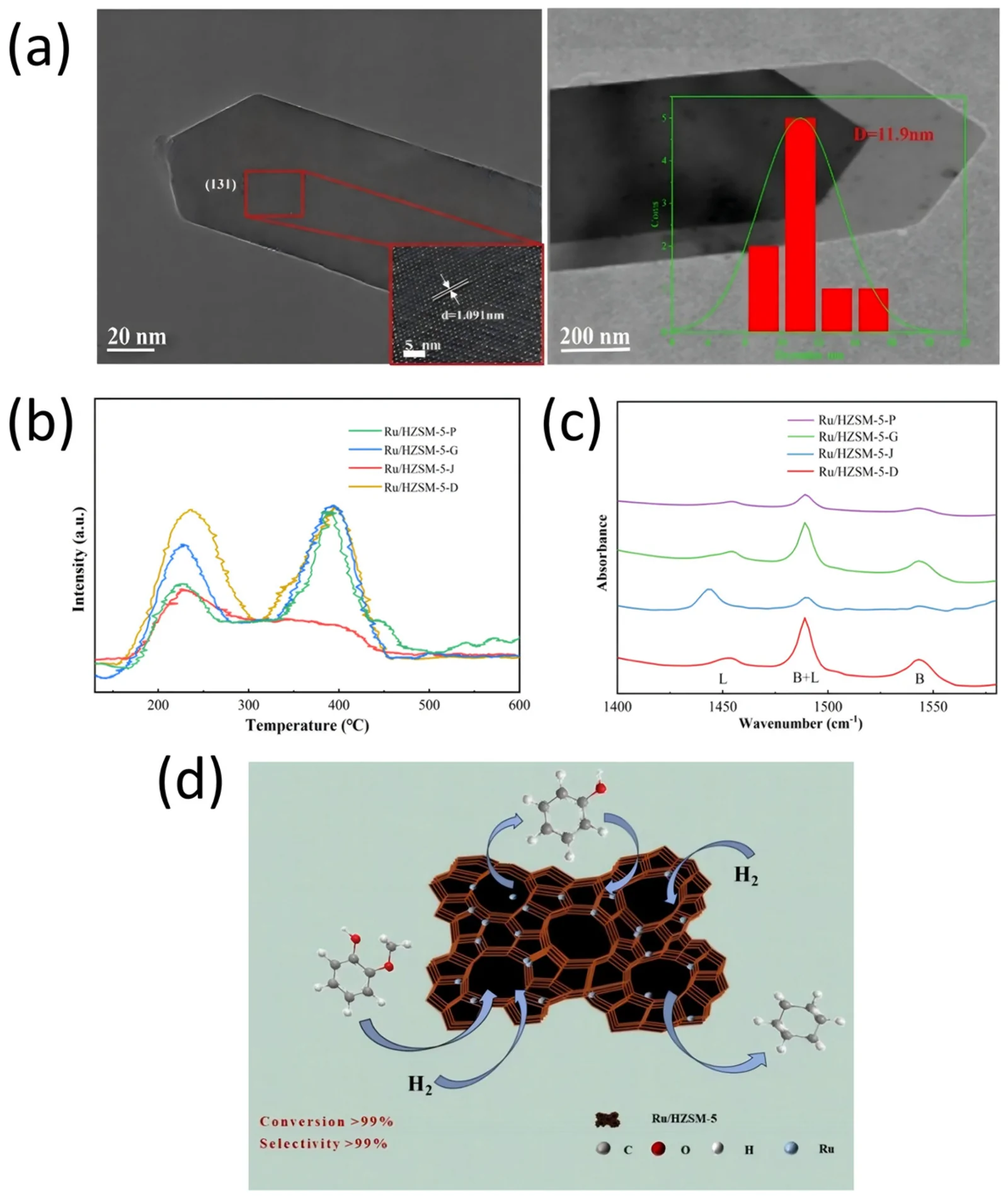
(**a**) TEM and TEM-mapping elemental spectra of Ru catalysts Ru/HZSM-5-P. Acidity analysis patterns of the four catalysts, Ru/HZSM-5-D, Ru/HZSM-5-J, Ru/HZSM-5-G, and Ru/HZSM-5-P: (**b**) NH_3_-TPD and (**c**) pyridine infrared. (**d**) Possible reaction mechanism of guaiacol degradation in the (Ru/HZSM-5-P)/*n*-hexane system. Reproduced with permission from [145]; published by American Chemical Society, 2024.

Beyond acid amount and acid strength, it is well established that the morphology and particle size of zeolites also significantly influence catalytic performance [160,161,162]. Tian et al. [145] conducted a comparative study on the effect of Ru/HZSM-5 catalysts with different morphologies on the selective HDO of guaiacol to cyclohexane. Among them, the Ru/HZSM-5-P catalyst with a two-dimensional nanosheet structure (Figure 14a) exhibited the best accessibility, smaller metal particle size (Figure 14b), favorable metal–support interaction, and appropriate B/L ratio (Figure 14c), which facilitated the activation of C-C and C-O bonds, thereby delivering the optimal conversion and cyclohexane selectivity. Mechanistic studies revealed that the metal and zeolite functioned synergistically in catalysis (Figure 14d): guaiacol diffused to the surface of the Ru/HZSM-5-P catalyst, where HZSM-5 provided interacting acid sites (both Brønsted and Lewis acid sites). Under a hydrogen atmosphere, the Ru active component dissociated H_2_ to generate active hydrogen species (H^+^), which attacked guaiacol, and cleaved the methoxy group to form phenol. Subsequently, Ru and H^+^ together facilitated the hydrogenation of the aromatic ring to yield cyclohexane [163,164].

In summary, metal–zeolite composite catalysts establish an efficient confined adjacency synergistic catalytic system in lignin HDO by integrating the hydrogen activation and hydrogenation capability of metal sites with the acid-site shape-selective catalytic function of zeolites. The advantages of this system are mainly manifested in three aspects: 1. Acid–metal bifunctional synergy. Metal sites are responsible for H_2_ dissociation and aromatic ring hydrogenation, while the Brønsted/Lewis acid sites of zeolites promote C-O bond cleavage and C-C bond isomerization/aromatization; the proximity effect between the two functionalities can significantly lower the reaction energy barrier. 2. Shape-selective catalysis and pore confinement. The well-defined pore structures facilitate the selective conversion of intermediates of specific molecular dimensions. 3. Tunable acidity. The acid amount and acid strength can be systematically modulated by adjusting the Si/Al ratio, enabling the establishment of a linear correlation between acidity and HDO activity of lignin, thereby providing a basis for the rational design of catalysts. Nevertheless, this system still faces several limitations: 1. Diffusion constraints. The microporous channels of zeolites pose mass-transfer limitations for macromolecular lignin and its oligomers, and are prone to coke deposition, leading to rapid catalyst deactivation [165]. 2. Insufficient hydrothermal stability. Under the hydrothermal conditions generated during the HDO reaction, the zeolite framework may undergo dealumination or collapse, compromising long-term operational stability [166]. Therefore, the development of hierarchical zeolites to improve mass transfer, along with the enhancement of hydrothermal stability, represents a crucial direction for advancing the practical application of metal–zeolite composite catalysts in the field of lignin-based aviation kerosene.

#### 2.4.2. Metal–Metal Oxide Composite Bifunctional Catalysts

In contrast to carbon materials or inert oxides (e.g., SiO_2_), which mainly rely on their specific surface area and pore structure to provide physical dispersion, metal oxides possess unique surface physicochemical properties, particularly oxygen vacancies and tunable SMSI, that enable deep synergy with the supported metal active sites. Consequently, metal oxides can actively participate in the reaction, modulating selectivity and stability during the HDO of lignin. Owing to these distinctive advantages, metal–metal oxide composite catalysts have become a research hotspot in the field of lignin HDO.

Studies have revealed that acidic supports such as Al_2_O_3_ and zeolite β, when used as catalyst carriers, are prone to rapid deactivation during HDO reactions due to coke formation [167]. In contrast, metal oxides, by virtue of the oxygen vacancies present in their bulk or on their surface, can lower the oxidation state of the corresponding metal, facilitating the adsorption of phenolic compounds and promoting C-O bond cleavage, thereby effectively enhancing catalytic activity [168,169,170]. Shu et al. [146] prepared three catalysts via a photochemical reduction method at room temperature, designated as Ru/TiO_2_, Ru/CeO_2_, and Ru/TiO_2_-CeO_2_. This approach utilized UV light to drive the in situ reduction of Ru^3+^ on the support surface, avoiding metal agglomeration typically caused by high-temperature calcination and achieving highly dispersed loading of Ru nanoparticles. HDO performance evaluation showed that Ru/TiO_2_-CeO_2_ could completely convert guaiacol to cyclohexane (with a selectivity of nearly 100%) at 230 °C and 1 MPa H_2_, significantly outperforming Ru/TiO_2_ (cyclohexane selectivity of 70.9%) and Ru/CeO_2_ (no deoxygenated products). This superior performance was attributed to the integration of the complementary advantages of TiO_2_ and CeO_2_ within the composite oxide support. The high specific surface area of TiO_2_ (203.77 m^2^/g) promoted the high dispersion of Ru, and H_2_-TPD along with TEM confirmed that Ru/TiO_2_-CeO_2_ possessed excellent hydrogen adsorption capacity, providing abundant active sites for aromatic ring hydrogenation. XPS analysis revealed that the introduction of CeO_2_ substantially increased the oxygen vacancy concentration, enhancing the adsorption of oxygenated substrates and the C-O bond activation capability of the catalyst. Adsorption experiments confirmed that Ru/TiO_2_-CeO_2_ exhibited significantly better adsorption of guaiacol compared to catalysts supported on single oxides. Meanwhile, NH_3_-TPD indicated that Ru/TiO_2_-CeO_2_ also contained abundant acid sites, providing sufficient acidic centers for C-O bond cleavage. The synergy among these three factors enables Ru/TiO_2_-CeO_2_ to achieve deep HDO of lignin under mild conditions, with a significant increase in the content of alkylphenols and hydrocarbons in the product.

**Figure 15:**
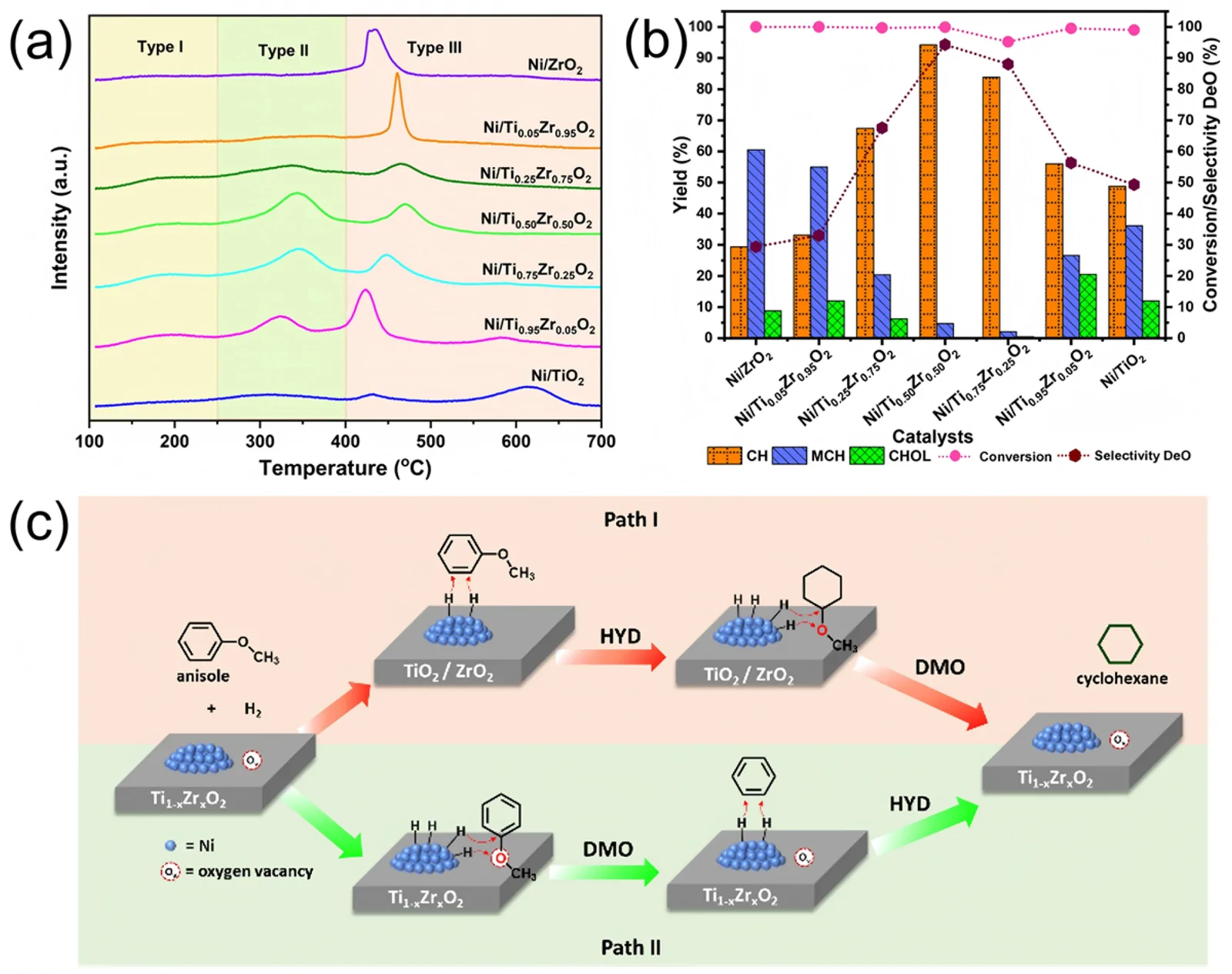
(**a**) O_2_-TPD profiles of Ni-supported mono and mixed oxide catalysts. (**b**) HDO of anisole with 3wt% Ni supported mono and mixed oxide catalysts. (**c**) A plausible mechanism for HDO of anisole cyclohexane with Ni/Ti_1−*x*_Zr*_x_*O_2_. Reproduced with permission from [147]; published by Royal Society of Chemistry, 2025.

Building upon the complementary advantages of composite oxide supports, Ali et al. [147] designed a series of Ni-doped Ti_1−*x*_Zr*_x_*O_2_ mixed oxide supports for the HDO of lignin model compounds. It was found that the incorporation of Zr into the TiO_2_ lattice induced the formation of abundant oxygen vacancies and Lewis acid sites, significantly enhancing the SMSI between Ni and the support (Figure 15a). Among them, the Ni/Ti_0.50_Zr_0.50_O_2_ catalyst achieved complete conversion of anisole with a cyclohexane yield of 94% at 230 °C and 4 MPa H_2_, whereas Ni/ZrO_2_ and Ni/TiO_2_ afforded cyclohexane yields of only 29% and 49%, respectively (Figure 15b). Multiple characterization techniques, including XPS, O_2_-TPD, NH_3_-DRIFTS, and H_2_-TPR, collectively elucidated the reaction mechanism (Figure 15c): the abundant oxygen vacancies in the mixed oxide support provided extensive adsorption sites for the activation of C-O bonds; the significant increase in both the number and strength of Lewis acid sites facilitated the adsorption of reactants onto oxygen vacancies via the oxygen atom of the methoxy group, thereby weakening the C-O bond; meanwhile, the SMSI promoted the generation of active hydrogen species at the metal–support interface. This synergy enables the efficient HDO of lignin-derived phenolics over low-loaded Ni catalysts under mild conditions, offering a catalyst design strategy for the synthesis of lignin-based jet-fuel-range cycloalkanes.

#### 2.4.3. Metal-Polyoxometalate (POM) Nanoclusters Composite Bifunctional Catalysts

Polyoxometalates (POMs) are a unique class of anionic polynuclear metal–oxygen clusters, exhibiting structural diversity at the atomic level. Countercations balance the negative charge on the framework; these cations may include protons serving as Brønsted acid sites, substituted metal cations, or metal ions with redox properties serving as Lewis acid sites. POMs with protons as the sole countercations are generally referred to as heteropoly acids (HPA) [171]. Owing to their tunable Brønsted/Lewis acidity and redox properties, POMs and POM-based composite materials have long been applied in heterogeneous catalysis [171,172,173]. Currently, most research has focused on Keggin-type POMs. The Keggin anion family has the general formula [XM_12_O_40_]^n−^ (where X = Si, P, etc.; M = Mo, W, etc.) [172]. Keggin-type polyoxometalates possess strong and stable acid sites, excellent redox properties, and remarkable stability [174], and have been employed in a wide range of biomass conversion processes [175] as well as in the HDO of various oxygenated compounds [176,177,178,179].

Metal NP-POM composites refer to either metal nanoparticles (NPs) supported on heterogeneous POMs, or metal NPs and POMs immobilized on an additional support. The intimate contact between the metal NPs and POMs facilitates the migration of substrates and intermediates over nanometer-scale distances between the metal sites and acid sites [180,181], thereby enabling synergistic effects in the catalytic HDO of lignin.

Kozhevnikov et al. reported that Pt catalysts supported on cesium salts of phosphotungstic acid could effectively catalyze the HDO of ethers [176] and ketones [182] under mild reaction conditions (<100 °C, <1 bar H_2_). Sautet and Yan demonstrated that polyoxometalate-stabilized Pt single-atom catalysts, owing to the effect of metal loading, could suppress the aggregation of Pt single atoms and weaken H_2_ adsorption energy, thereby exhibiting a lower apparent reaction energy barrier in hydrogenation reactions [178]. Recently, Liu and co-workers found that the combination of Pt/C and polyoxometallic acids enabled the upgrading of lignin bio-oil to hydrocarbons under mild aqueous conditions, wherein the polyoxometallic acid acted as both an H_2_ buffer and a support. However, the good water solubility of polyoxometallic acids may hinder their recovery process [148].

**Figure 16:**
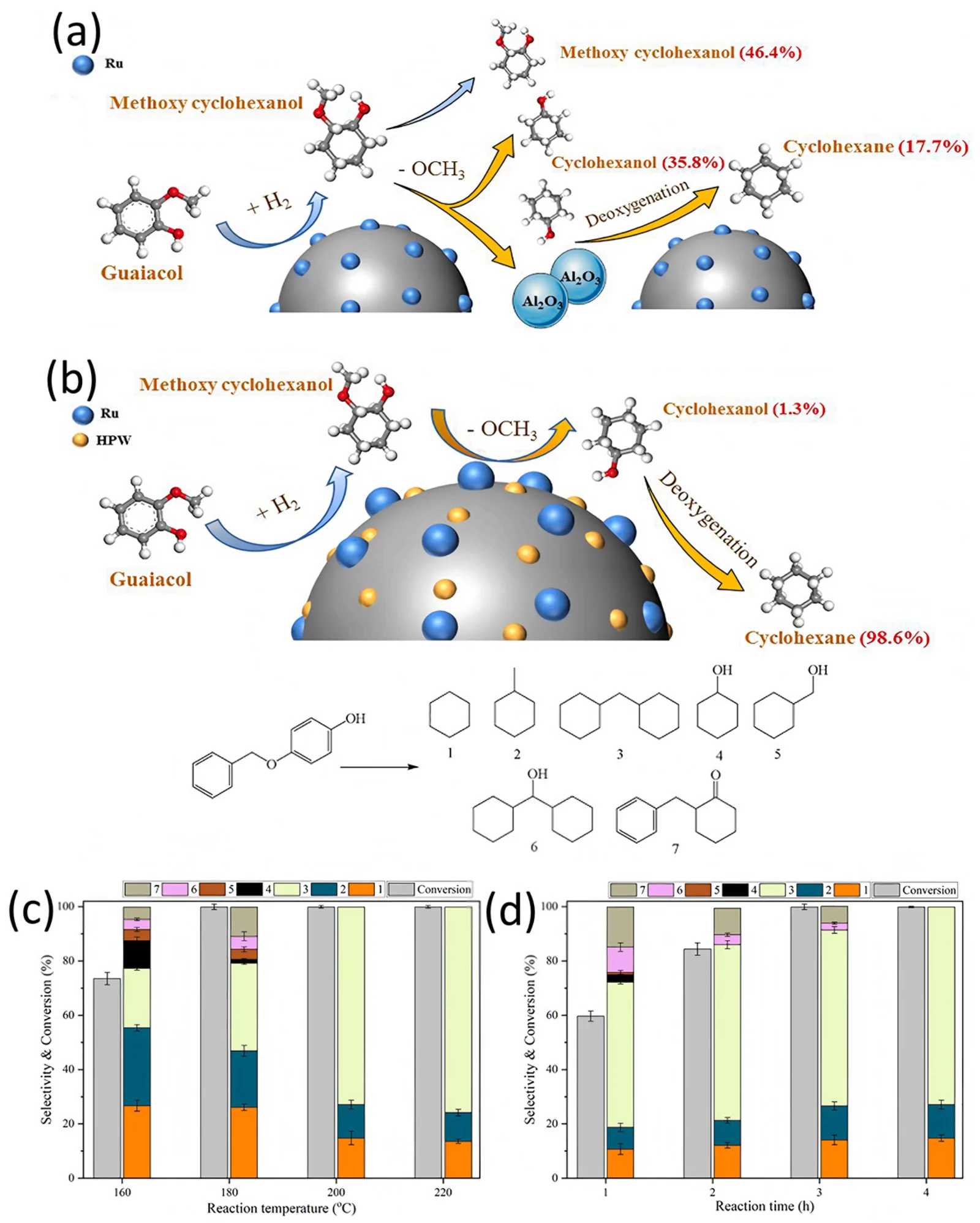
The synergistic effect of active metal–acid sites and the guaiacol HDO reaction pathway (**a**) Ru/C-Al_2_O_3_ (**b**) Ru/C-HPW. Effects of (**c**) reaction temperature and (**d**) reaction time on the 4-benzyloxy phenol HDO. Reproduced with permission from [137]; published by Elsevier, 2022.

To maximize the synergistic effect, Yang et al. [137] designed a bifunctional catalyst system composed of Ru/C and phosphotungstic acid (HPW), designated as Ru/C-HPW, for the HDO of lignin-derived phenolic compounds. This system cleverly exploited the temperature-controlled phase-transfer behavior of HPW, which is solid at room temperature and melts into a liquid phase upon heating to the reaction temperature (200 °C), thereby enabling uniform dispersion and adsorption onto the Ru/C surface and achieving intimate contact between the metal sites (Ru) and acid sites (HPW) in the liquid phase. This “in-situ assembly” strategy integrated catalyst preparation with the HDO reaction in a single step, avoiding the issue of poor dispersion between metal and acid components typically encountered in conventional methods such as wet impregnation and co-precipitation. The intimate metal–acid contact significantly enhanced the synergistic effect: guaiacol first underwent aromatic ring hydrogenation at Ru sites to form methoxycyclohexanol, followed by efficient C-O bond cleavage catalyzed by the Lewis acid sites of HPW; the deoxygenated product then underwent a second hydrogenation at Ru sites to yield cyclohexane (Figure 16a,b). Benefiting from the efficient mass transfer of substrates between the two types of sites, guaiacol achieved complete conversion within 4 h at 200 °C and 1 MPa H_2_, with a cyclohexane selectivity as high as 98.6%. Moreover, the reaction rate far surpassed that of solid acid systems such as Ru/C-Al_2_O_3_, which suffered from limited mass transfer across the solid–solid interface and afforded a cyclohexane selectivity of only 46.4% (Figure 16c,d). The catalyst showed no significant loss of activity after five cycles, and the use of HPW avoided the risk of water pollution associated with liquid acids such as H_3_PO_4_. In addition to guaiacol, this system also exhibited excellent HDO performance toward real lignin oil, increasing the hydrocarbon content from 5.5% to 78.9%. This work demonstrates the unique advantages of the temperature-controlled phase-transfer strategy in constructing efficient metal–acid bifunctional catalytic systems, and provides a feasible route for the mild synthesis of lignin-based cycloalkanes.

As mentioned above, the HDO of lignin under low temperature and low pressure is constrained by the high activation energy required for C-O bond cleavage and the extremely low solubility of hydrogen in the aqueous phase. Liu et al. [148] constructed a synergistic bifunctional three-phase hydrogen transfer catalytic system (SiW_12_-Pt/C) composed of the Keggin-type polyoxometallic acid H_4_SiW_12_O_40_ and suspended Pt/C particles, which effectively avoided the aforementioned issues. By exploiting the low redox potential of H_4_SiW_12_O_40_ (E° = –0.05 V vs. NHE), the authors achieved a reversible redox reaction with H_2_ in the presence of Pt/C, enabling the rapid transfer of H_2_ from the gas phase to the liquid phase and its release as H^+^. This effectively raised the concentration of active hydrogen species in the solution to a level equivalent to that of dissolved hydrogen under 10 atm of H_2_ pressure. Meanwhile, H_4_SiW_12_O_40_, acting as a superacid, could protonate partially hydrogenated phenolic intermediates to form oxonium ions, substantially reducing the activation energy for C-O bond cleavage. Under this bifunctional synergy, the SiW_12_-Pt/C catalyst achieved HDO of phenol at 85 °C with an H_2_ partial pressure of only 0.64 atm, affording a total hydrocarbon (benzene and cyclohexane) yield of 90.8%. Other lignin-derived phenolics and dimeric model compounds, such as guaiacol, also underwent efficient HDO under temperatures below 100 °C and atmospheric H_2_, with hydrocarbon yields in the range of 60–90%. This work, through the “hydrogen buffer-superacid” bifunctional design of polyoxometalates, overcomes the reliance on high temperature and high pressure in traditional HDO, and offers a new strategy for the mild synthesis of lignin-based aromatics and cycloalkanes.

However, the use of metal NP-POM catalysts for biomass valorization still faces considerable challenges. First, the leaching of active components remains a common issue that compromises catalyst stability. In addition, most of the reported POMs for biomass utilization have focused on Keggin-type structures, while the application potential of other architectures, such as Wells–Dawson- and Lindqvist-type POMs, remains largely unexplored. Future research should aim to gain a deeper understanding of the relationships between the catalytic performance and the chemical/electronic/structural properties of metal NP-POMs-based catalysts, which is of great significance for the efficient HDO of lignin [175].

#### 2.4.4. Metal–Other Support Composite Bifunctional Catalysts

Beyond the mainstream composite catalyst systems, including metal–zeolite, metal–oxide, and metal–POM combinations, other supports, exemplified by carbon-based materials and metal–organic frameworks (MOFs), have also demonstrated significant potential in the field of lignin HDO, owing to their unique physicochemical properties.

**Figure 17:**
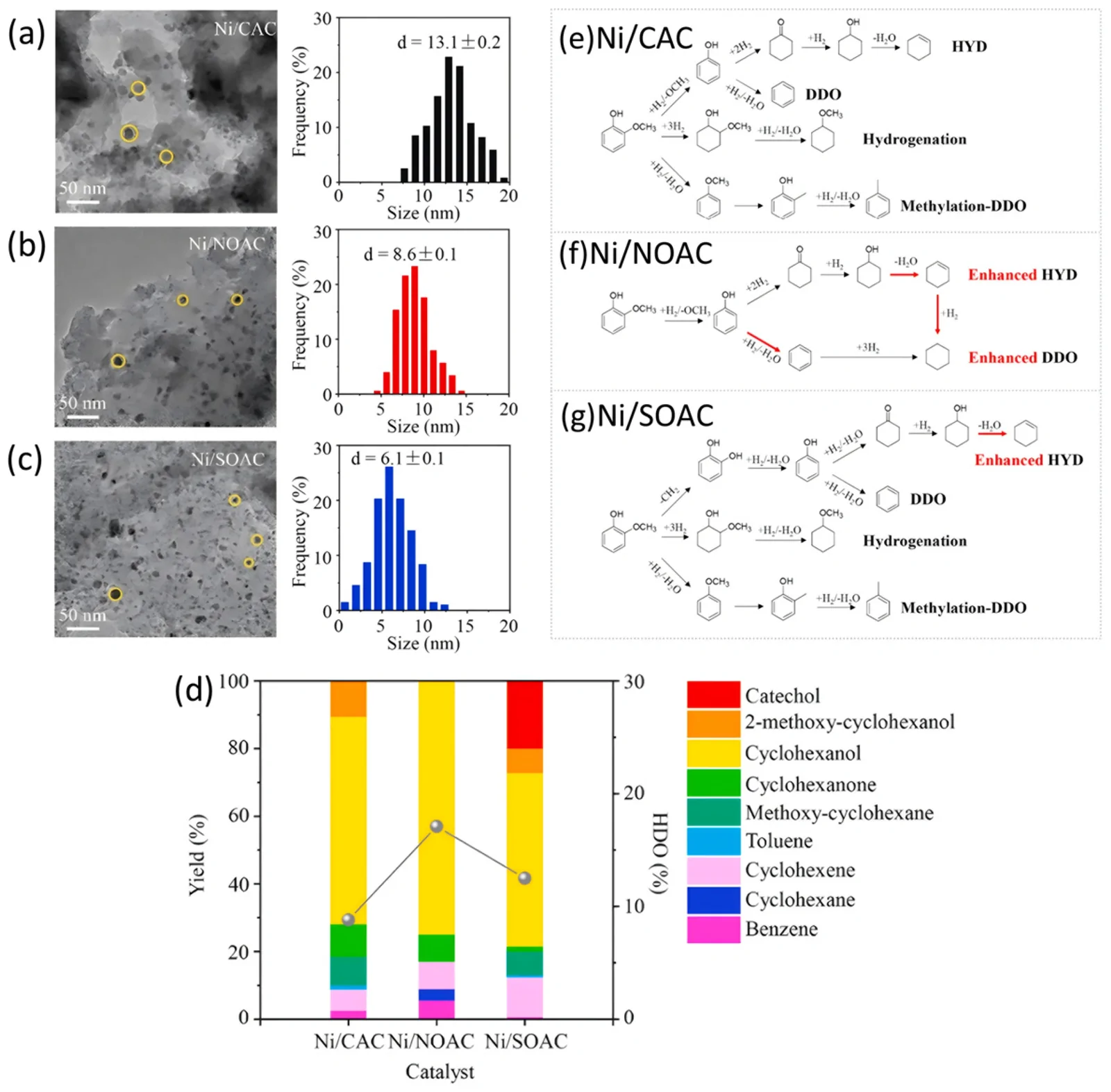
HRTEM images of (**a**) Ni/CAC; (**b**) Ni/NOAC; (**c**) Ni/SOAC and the corresponding Ni particle size normal distribution. (**d**) Guaiacol HDO result over Ni/CAC, Ni/NOAC and Ni/SOAC catalysts. Proposed reaction routes of guaiacol on Ni/CAC (**e**), Ni/NOAC (**f**) and Ni/SOAC (**g**) catalysts. Note: The thick red line represents that the reaction route is promoted by the modified surface chemistry. Reproduced with permission from [149]; published by Elsevier, 2022.

Activated carbon is frequently employed as a catalyst support owing to its unique physicochemical properties, such as the presence of oxygen-containing functional groups on its surface, a high micropore or mesopore surface area, and structural and morphological stability at elevated temperatures and in various liquid media [183,184]. Consequently, compared to acidic supports with poor water resistance, such as Al_2_O_3_ and zeolites [185,186,187], which are widely used in the HDO of lignin and its derivatives [188,189], activated carbon is more suitable for the HDO of lignin derivatives in alkaline solutions. Xu et al. [149] investigated three nickel catalysts supported on coconut-shell-derived activated carbon for the HDO of guaiacol under alkaline conditions. To enhance the surface acidity of the catalyst, the authors pretreated the activated carbon with HNO_3_ and H_2_SO_4_ to obtain Ni/NOAC and Ni/SOAC, respectively, while the catalyst without acid pretreatment was designated as Ni/CAC. Boehm titration, FT-IR, and XPS characterization revealed that acid pretreatment significantly increased the content of acidic oxygen-containing functional groups on the support surface. These acidic groups played a dual role: on one hand, they directly promoted the cleavage of C-O bonds in guaiacol as Brønsted acid sites; on the other hand, they enhanced the electrostatic adsorption of Ni^2+^ precursors, reducing the average particle size of the resulting Ni nanoparticles from 13.1 nm in Ni/CAC to 8.6 nm in Ni/NOAC and 6.1 nm in Ni/SOAC (Figure 17a–c). Under alkaline conditions at 300 °C and 4 MPa H_2_, the HDO efficiency of Ni/NOAC (17.1%) was approximately twice that of Ni/CAC (8.8%), with significantly improved yields of benzene and cyclohexene. In contrast, the cyclohexene yield over Ni/SOAC was nearly double that over Ni/CAC, while the yields of benzene and toluene were lower, which was attributed to the rapid hydrogenation saturation of the aromatic ring catalyzed by Ni/SOAC (Figure 17d). Based on these results, the reaction pathways were elucidated: following acid pretreatment, the smaller Ni particle size provided more metal active sites for aromatic ring hydrogenation, while the abundant acid sites accelerated C-O bond cleavage, thus promoting both the direct deoxygenation (DDO) and hydrogenation–deoxygenation (HYD) routes simultaneously (Figure 17e,f). This work reveals the structure–activity mechanism by which the surface chemistry of carbon supports synergistically enhances HDO performance through the modulation of metal dispersion and acid site density, providing a catalyst design strategy for the efficient valorization of lignin components under strongly alkaline conditions.

As mentioned above, the key challenge in the catalytic HDO of lignin is to achieve rapid and selective cleavage of aromatic C-O bonds by hydrogen, while avoiding the hydrogenation of aromatic C=C bonds [190]. Therefore, the selective cleavage of aromatic C-O bonds requires the combination of metal sites for H_2_ activation and Lewis acid sites of bifunctional catalysts for substrate adsorption. Among the various acidic supports employed for biomass upgrading, MOF materials have been utilized for the selective catalytic cleavage of C-O bonds, owing to their exceptional structural properties [191,192,193]. Specifically, the unsaturated metal ions within the MOF framework exhibit Lewis acidity, which can be exploited for the adsorption of lignin-derived phenolic compounds [192,194,195]. UiO-66, a MOF characterized by a high specific surface area, tunable pore structures, ease of scale-up, and thermal stability [196,197], has been applied in heterogeneous catalysis [194,195]. Typically, two types of defects exist in the UiO-66 framework, namely missing-cluster defects and missing-linker defects [197,198]. Both types serve as open Lewis acid sites within the UiO-66 structure, which are crucial for catalytic biomass upgrading [197,199]. Accordingly, Phan et al. [150] synthesized a series of UiO-66 supports with varying linker defects (UiO-66-ydef) via a microwave-assisted solvothermal method, and subsequently loaded Pt by impregnation to obtain Pt/defective UiO-66 catalysts. By introducing isophthalic acid (IPA) as a mixed linker, the partial absence of IPA exposed more open Lewis acid sites on the Zr_6_O_4_(OH)_4_ clusters, while the linker defects also introduced additional hydroxyl groups, which meant an increase in Brønsted acid sites. NH_3_-TPD and XPS characterization revealed that the defective UiO-66 samples exhibited a total acid amount 1.5–2.5 times higher than that of pristine UiO-66, with the number of linker defects increasing with IPA content. CO chemisorption and TEM confirmed that the introduction of defects did not significantly alter the Pt dispersion (~22%) or particle size (3.5 nm); hence, the enhanced catalytic performance was primarily attributable to the increased acidity of the support rather than geometric changes of the metal sites. Under continuous fixed-bed reaction conditions at 270 °C and 1 MPa, the 3Pt/UiO-66-20def catalyst achieved an anisole HDO conversion of 86%, with a TOF 2–3 times higher than that of pristine 3Pt/UiO-66. In terms of selectivity, the selectivity toward benzene increased substantially as the defect content rose from 0 to 20%, with the TOF based on benzene yield increasing by more than 10-fold. This indicated a significant shift in the reaction pathway: the pristine catalyst, owing to the strong hydrogenation capability of Pt sites, tended to convert anisole to cyclohexane through aromatic ring hydrogenation, whereas the abundant Lewis acid sites on defective UiO-66 preferentially adsorbed and activated the oxygen atom of the methoxy group, steering the reaction toward the demethoxylation pathway and thereby converting the oxygenated aromatic ring to benzene. However, 3Pt/UiO-66-30def exhibited decreased activity due to coke formation caused by excessively strong acid sites, indicating that an appropriate defect concentration is key to achieving a balance between high activity and stability. This work reveals the design strategy of defect engineering in MOF supports to steer HDO reaction pathways by modulating the type and density of acid sites, offering a new approach for the selective synthesis of lignin-based aromatics.

Beyond conventional supports such as zeolites and metal oxides, other supports, including carbon-based materials and MOFs, have opened up new avenues for the design of catalytic systems for lignin HDO, owing to their highly tunable physicochemical properties. Nevertheless, both types of supports face common challenges: the surface functional groups of carbon-based materials are susceptible to loss under high-temperature reduction conditions [200], while the microporous structures of MOFs impose significant mass-transfer limitations for macromolecular substrates [201].

In bifunctional catalysts, cascade deoxygenation proceeds through the cooperation of metal sites for H_2_ activation with acid, redox, or defect sites for C-O cleavage. The proximity and ratio between these two functions prove decisive: too few acid sites limit deoxygenation, while excessive acidity promotes coke formation and over-hydrogenation. Metal–zeolite systems benefit from tunable Brønsted/Lewis acidity and shape selectivity [144,145], while metal–oxide systems exploit oxygen vacancies and SMSI to activate C-O bonds and stabilize intermediates [146,147]. Metal–POM systems demonstrate that temperature-controlled phase transfer can maximize metal-acid contact and mass transfer [137], and carbon- or MOF-supported systems show that surface chemistry and defect engineering can steer reaction pathways [149,150]. Future research should focus on the construction of hierarchical pore structures to improve mass transfer, the rational regulation of synthesis conditions to enhance support–metal synergistic stability, and the development of adaptive catalytic systems with high selectivity toward target products by exploiting the structural tunability of these materials, thereby advancing the directed synthesis of lignin-based jet fuel components toward practical application.

### 2.5. Single-Atom Catalysts

Compared with traditional nanoparticle and nanocluster catalysts, single-atom catalysts (SACs) have attracted widespread attention in energy- and environment-related applications, owing to their high atom utilization efficiency, unique electronic states, and well-defined active sites [202,203,204]. More importantly, by virtue of their uniform active sites and well-defined coordination structures, SACs tend to steer the lignin conversion pathway toward a single route, thereby affording high selectivity. For example, single-atom catalysts such as Ru/CeO_2_ [205], Pt-Fe/CeO_2_ [206], and Au_1_/Nb_2_O_5_ [207] have been shown to effectively catalyze the cleavage of C-O bonds in lignin models or lignin itself, yielding cycloalkanes in high yields. Table 5 summarizes recent studies on the application of single-atom catalysts in the HDO of lignin and its derivatives.

**Figure 18:**
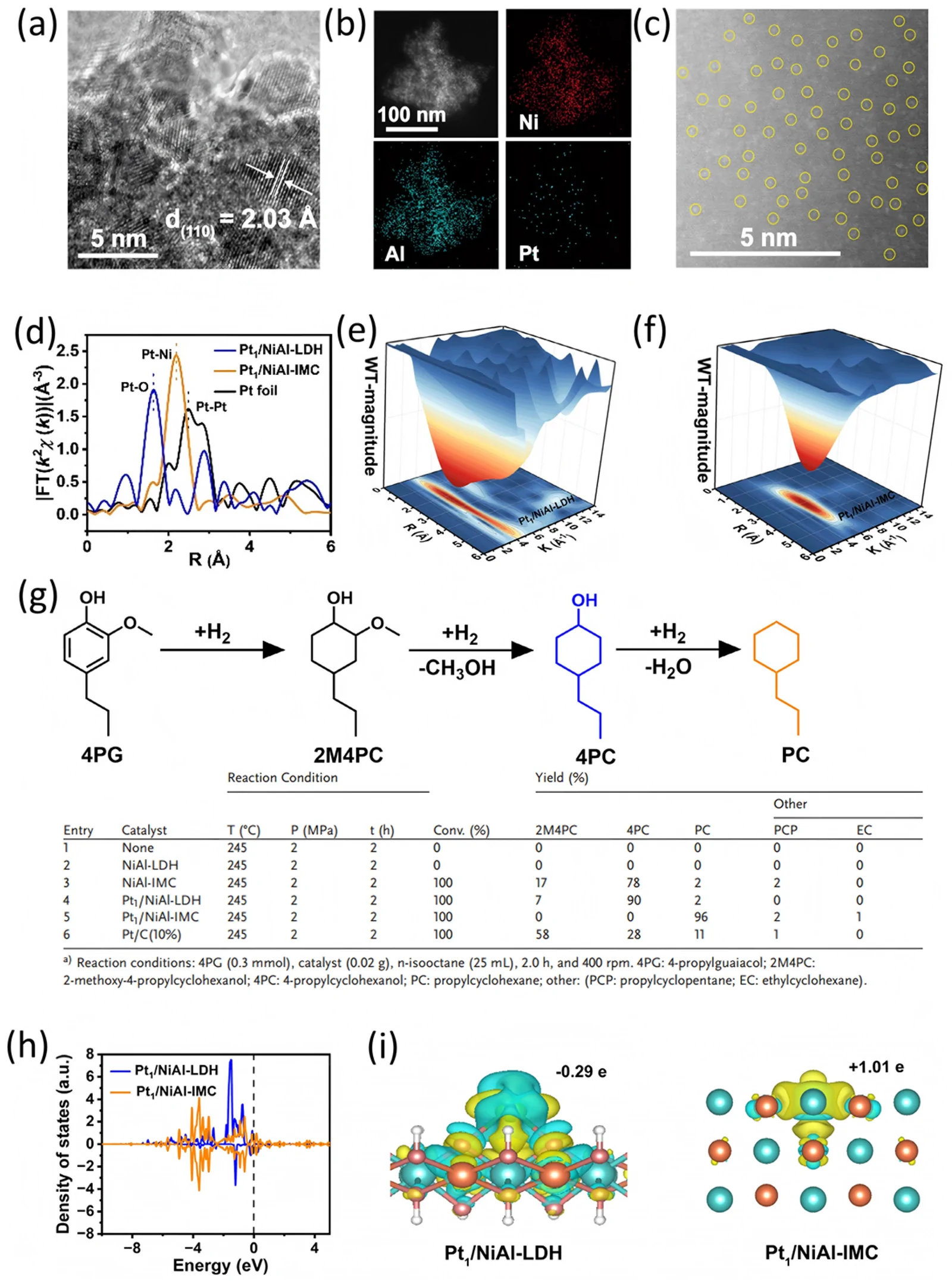
Synthesis, characterization and performance testing of Pt SAC. (**a**) HRTEM image, (**b**) EDX spectrum analysis and (**c**) AC-HAADF-STEM image of Pt_1_/NiAl-IMC. (**d**) FT-EXAFS spectra of Pt_1_/NiAl-LDH, Pt_1_/NiAl-IMC, and Pt foil at the Pt K-edge samples. WT-EXAFS of (**e**) Pt_1_/NiAl-LDH and (**f**) Pt_1_/NiAl-IMC samples. (**g**) Reaction of 4-Propylguaiacol over different catalyst. (**h**) PDOS of Pt d orbital in Pt_1_/NiAl-LDH and Pt_1_/NiAl-IMC samples. The gray dotted line denotes the Fermi level. (**i**) Calculated charge density difference distribution of Pt_1_/NiAl-LDH and Pt_1_/NiAl-IMC samples. Reproduced with permission from [208]; published by John Wiley and Sons, 2025.

However, the coordination structures of SACs are typically “locked” under specific synthesis conditions, making it difficult to achieve selective switching between different products at the same metal center once the structure is formed. Charge density, as a key electronic parameter governing catalytic behavior, has been recognized as an effective means to overcome this limitation through its precise regulation. Nevertheless, how to achieve controllable modulation of charge density at single-atom sites and establish a quantitative correlation with product selectivity remains a formidable challenge. Dai et al. [208] employed a topotactic transformation strategy using NiAl layered double hydroxide (LDH) precursors to construct two Pt single-atom catalysts with distinct local electronic structures by tuning the reduction temperature (Figure 18a–c). In Pt_1_/NiAl-LDH, obtained by reduction at 200 °C, Pt was anchored on the LDH surface with Pt-O*_x_* coordination, exhibiting an electron-deficient state. In contrast, in Pt_1_/NiAl-IMC, obtained by reduction at 400 °C, the interlayer species of LDH were removed and converted into NiAl intermetallic compounds, with Pt existing in a Pt-Ni*_x_* coordination configuration. Electron transfer between Pt and the NiAl substrate rendered Pt in an electron-rich state (Figure 18d–f). XANES, XPS, and DFT calculations collectively confirmed the completely opposite charge density characteristics of the two Pt sites. In the HDO of 4-propylguaiacol, Pt_1_/NiAl-LDH selectively catalyzed aromatic ring hydrogenation and demethoxylation, affording 4-propylcyclohexanol with >90% selectivity. In contrast, Pt_1_/NiAl-IMC further enabled complete cleavage of the C-OH bond, yielding propylcyclohexane with 96% selectivity (Figure 18g). Mechanistic studies revealed that the electron-deficient Pt sites on Pt_1_/NiAl-LDH exhibited excessively strong adsorption toward 4-propylcyclohexanol, causing the reaction to stall at the alcohol product, whereas the electron-rich Pt sites on Pt_1_/NiAl-IMC, through d-electron back-donation into the π* antibonding orbital of the C-O bond, weakened the C-O bond energy and thereby drove complete deoxygenation (Figure 18h,i). This strategy proved equally effective with real lignin feedstocks, achieving a cycloalkane yield of 25.5 wt%. This work demonstrates the precise switching of product selectivity at the same metal center through charge density modulation of single-atom sites via different support structures, providing an atomic-scale catalyst design paradigm for the directed synthesis of lignin-based jet fuel components.

As mentioned above, Keggin-type polyoxometalates possess abundant and strong acid sites, and have therefore been frequently employed to stabilize and disperse metal atoms [176,178,182]. However, the high water solubility of polyoxometalates renders them susceptible to leaching in aqueous phases [175]. To address this issue, Gao et al. [209] introduced Cs^+^ and employed cesium salts of Keggin-type polyoxometalates as supports, constructing an atomically dispersed Pt catalyst (Pt_0.4_/CsPW-H_2_) via impregnation followed by hydrogen reduction. This approach converted the heteropoly acid into an insoluble cesium salt while retaining the strong acidity of the Keggin structure. XAS and HAADF-STEM confirmed that Pt atoms were anchored as single atoms at the bridging oxygen sites of the [PW_12_O_40_]^3−^ framework, with a Pt-O coordination number of approximately 2. NH_3_-TPD and H_2_-TPD characterization revealed that the catalyst possessed both strong acidity and high hydrogen storage capacity, and DFT calculations further confirmed the correlation between its structure and catalytic activity. Benefiting from these structural advantages, Pt_0.4_/CsPW-H_2_ achieved efficient HDO of various lignin-derived phenolic monomers and β-O-4 dimers under mild conditions at 150 °C and 0.1–2 MPa H_2_, with a substrate/Pt molar ratio as high as 909 and a propylcyclohexane yield of up to 97%. Over 20 consecutive cycles, the total turnover number reached 15,095 mol_propylcyclohexane_ mol_Pt_^−1^. When applied to the direct conversion of eucalyptus reductive catalytic fractionation (RCF) lignin oil under solvent-free conditions, the catalyst afforded 32.7wt% of C6-C10 cycloalkanes with a degree of deoxygenation of 95%. This work demonstrates a design strategy combining single-atom dispersed Pt sites with polyoxometalate synergy, offering a new route for the mild synthesis of lignin-based cycloalkanes.

Although single-atom catalysts can achieve high selectivity through atomically dispersed metal sites, how to realize efficient C-O bond cleavage on non-noble metal single atoms remains a significant challenge in this field. Transition metal carbides, such as β-Mo_2_C, are considered promising supports for single atoms owing to their noble-metal-like electronic properties and oxygen affinity, which enable the modulation of charge density and adsorption behavior of single atoms through metal–support interactions [211,212,213]. Guo et al. [111] employed a co-precipitation method combined with temperature-programmed carburization to prepare a catalyst (Ni_1_/β-Mo_2_C) featuring Ni single atoms dispersed on a β-Mo_2_C support, and applied it to the HDO of dihydroeugenol (DHE) to produce propylcyclohexane. Under conditions of 260 °C and 4 MPa H_2_, this catalyst achieved nearly 100% conversion of DHE and nearly 100% selectivity toward propylcyclohexane. HAADF-STEM and XAFS confirmed that Ni atoms were anchored as single atoms on the support surface via Ni-C3 coordination. XPS and charge density analysis revealed electron transfer from Mo to Ni, rendering the Ni sites electron-rich and optimizing the adsorption behavior of reactants. DFT calculations further disclosed that the Ni single-atom sites altered the adsorption geometry of intermediates from perpendicular to parallel orientation, thereby lowering the energy barrier for aromatic ring hydrogenation and shifting the reaction pathway from preferential deoxygenation to a synergistic hydrogenation-deoxygenation mode, ultimately affording propylcyclohexane with high selectivity. In addition, the catalyst exhibited good substrate generality toward various lignin-derived phenolics and maintained high activity after recycling. This work provides an atomic-scale design strategy for the selective conversion of lignin derivatives into jet-fuel-range cycloalkanes using non-noble metal single-atom catalysts.

In addition, cationic vacancies also serve as effective anchoring sites for stabilizing single atoms, owing to their unique electronic effects at the microscopic scale [214,215]. Meanwhile, LDHs with their distinctive lattice effects that enable ordered dispersion of metals, are ideal precursors for constructing cationic vacancies [216,217]. Hu et al. [210] employed a topotactic transformation strategy based on LDH precursors to construct NiFe-LDH cationic vacancies via alkaline etching, followed by impregnation and reduction to anchor Mo single atoms within the vacancies, yielding the Mo/NiFe_71_-V single-atom catalyst. AC-HAADF-STEM and EXAFS confirmed that Mo atoms occupied the cationic vacancies of the NiFe matrix as isolated single atoms, forming a Mo-Ni coordination structure. XPS, XANES, and charge density difference analyses further revealed that electron transfer occurred from the anchored Mo to the adjacent Ni, rendering the Mo sites electron-deficient. DFT calculations showed that the adsorption energy of cyclohexanol on the vacancy-anchored Mo sites was as low as −1.16 eV, indicating that the vacancy-induced metal–metal interaction significantly enhanced the catalyst’s adsorption capacity toward oxygenated intermediates. Benefiting from this, the catalyst achieved nearly 100% conversion and a cyclohexane yield of 99.5% in the HDO of guaiacol at 240 °C and 4 MPa H_2_. Moreover, as the regeneration of active sites required overcoming an energy barrier of only 0.10 eV, the catalyst maintained a yield of 92.1% after six cycles, showcasing superior high-temperature stability compared to most reported single-atom systems. In addition, the catalyst exhibited excellent selectivity toward cycloalkanes for various real lignin feedstocks. To sum up, by utilizing cationic vacancies to stabilize non-noble metal single-atom catalysts, this work provides a new strategy for the efficient and stable production of lignin-based cycloalkanes.

Over the past few decades, SACs have been widely employed in catalytic reactions owing to their uniform active sites and near-100% atom utilization efficiency [218,219,220]. Moreover, their catalytic behavior is governed by the coordination environment, charge density, and anchoring site rather than by size alone [208]. However, the weak adsorption capability of isolated metal atoms toward hydrogen donor molecules, coupled with their inability to effectively stabilize intermediates after dehydrogenation, limits the generation of active hydrogen species. Consequently, non-noble metal SACs often fail to achieve satisfactory catalytic performance in C-O bond cleavage reactions [221,222]. Furthermore, SACs typically possess relatively stable coordination structures under specific synthesis conditions, making the modulation of catalytic selectivity through atomic microenvironment regulation a challenging goal, thereby restricting their potential for efficient lignin conversion [223,224,225]. Therefore, metal nanoparticles remain the mainstream catalysts in lignin HDO reactions [226].

The nanocatalysts discussed above have already demonstrated the ability to achieve highly selective C-O bond cleavage and controlled aromatic ring hydrogenation in the HDO of lignin model compounds. Although the foregoing discussion has centered on well-defined model compounds, several of the catalysts surveyed above have also been extended to lignin-derived oils and even to raw lignin. For instance, Mo-Co_9_S_8_/Al_2_O_3_ converted not only model substrates but also lignin bio-oil and raw lignin into aromatic hydrocarbons [69]; Ni_2_P-Al_2_O_3_ proved effective for the conversion of lignin oil and poplar lignin [113]; and Ru/C-HPW increased the hydrocarbon content of a real lignin oil from 5.5% to 78.9% [137]. Likewise, among single-atom systems, Pt_1_/NiAl-IMC afforded a 25.5wt% cycloalkane yield from real lignin feedstocks [208], and Pt_0.4_/CsPW-H_2_ converted eucalyptus RCF lignin oil into 32.7wt% of C6-C10 cycloalkanes with a deoxygenation degree of 95% [209]. These studies confirm that the catalytic principles established on model substrates are, in a qualitative sense, transferable to real lignin: the same active sites that mediate selective C-O bond cleavage and controlled aromatic-ring hydrogenation remain operative.

Nevertheless, the results obtained on real lignin are not fully comparable to those on model compounds, which generally deliver higher hydrocarbon yields and selectivities than those achieved on genuine substrates. The principal reason is that real lignin possesses a highly cross-linked macromolecular structure, complex functional group compositions, and significant feedstock heterogeneity [1,29,227]. Its conversion to jet-fuel-range hydrocarbons is not a single HDO process, but rather involves multiple consecutive steps, including lignin depolymerization, stabilization of reactive intermediates, deoxygenation, and selective aromatic ring hydrogenation [227,228]. Different steps impose distinct requirements on catalytic function and reaction conditions: lignin depolymerization generally demands strong bond activation capability and relatively high temperatures, whereas the subsequent HDO requires efficient hydrogen activation and transfer, along with a precise balance between C-O bond cleavage and aromatic ring saturation. Meanwhile, if the reactive intermediates generated during depolymerization cannot be stabilized in a timely manner, they are prone to repolymerization, condensation, and coke deposition, leading to carbon loss and catalyst deactivation [203,227]. Therefore, the core issue in the cascade conversion of real lignin is not simply the addition of more reaction steps, but rather how to coordinate the functions of depolymerization, stabilization, and HDO through the composition, active sites, and spatial organization of the catalyst.

## 3. Catalyst Design in Cascade Conversion Processes

On the basis of the multifunctional catalytic requirements for the cascade conversion of real lignin identified above, the existing cascade systems have mainly developed two catalyst design paradigms. The first is a two-stage cascade configuration, in which depolymerization and HDO are separately assigned to different reaction units, with catalysts tailored to each step, thereby achieving macroscopic decoupling of catalytic functions and reaction conditions. The second is simultaneous depolymerization and HDO (one-pot synthesis), which involves constructing bifunctional or multicomponent nanocatalysts within a single reactor, enabling in situ stabilization of depolymerization intermediates and continuous deoxygenation through the proximity synergy between acid sites and metal sites, as well as hydrogen spillover. In the following sections, these two types of cascade systems will be discussed from the perspectives of catalyst functional design and structure-activity relationships.

Fang et al. [229] constructed a two-stage cascade system by synthesizing a dual-catalyst combination comprising W*_x_*C@CS and Ni/CNT, which allocated C-O bond cleavage and aromatic ring hydrogenation to different active components. W*_x_*C@CS, with a surface C/W ratio of approximately 7–8, primarily promoted the cleavage of aryl C-O bonds, converting guaiacol into phenolic intermediates, while Ni/CNT was responsible for H_2_ activation and aromatic ring hydrogenation. The resulting cyclohexanol was then further deoxygenated at either W*_x_*C or Ni sites. The intimate contact between the two catalysts shortened the intermediate transfer distance and reduced the apparent activation energy of the system to 56.7 kJ mol^−1^. The Ni particle size also influenced product selectivity: smaller Ni particles favored deep deoxygenation to cyclohexane, whereas larger particles were more conducive to the retention of cyclohexanol. This system achieved a guaiacol conversion of nearly 100% and a cyclohexane selectivity of 92.4%, underscoring the critical roles of active-site functional separation, particle size regulation, and spatial synergy in the design of cascade catalysts.

**Figure 19:**
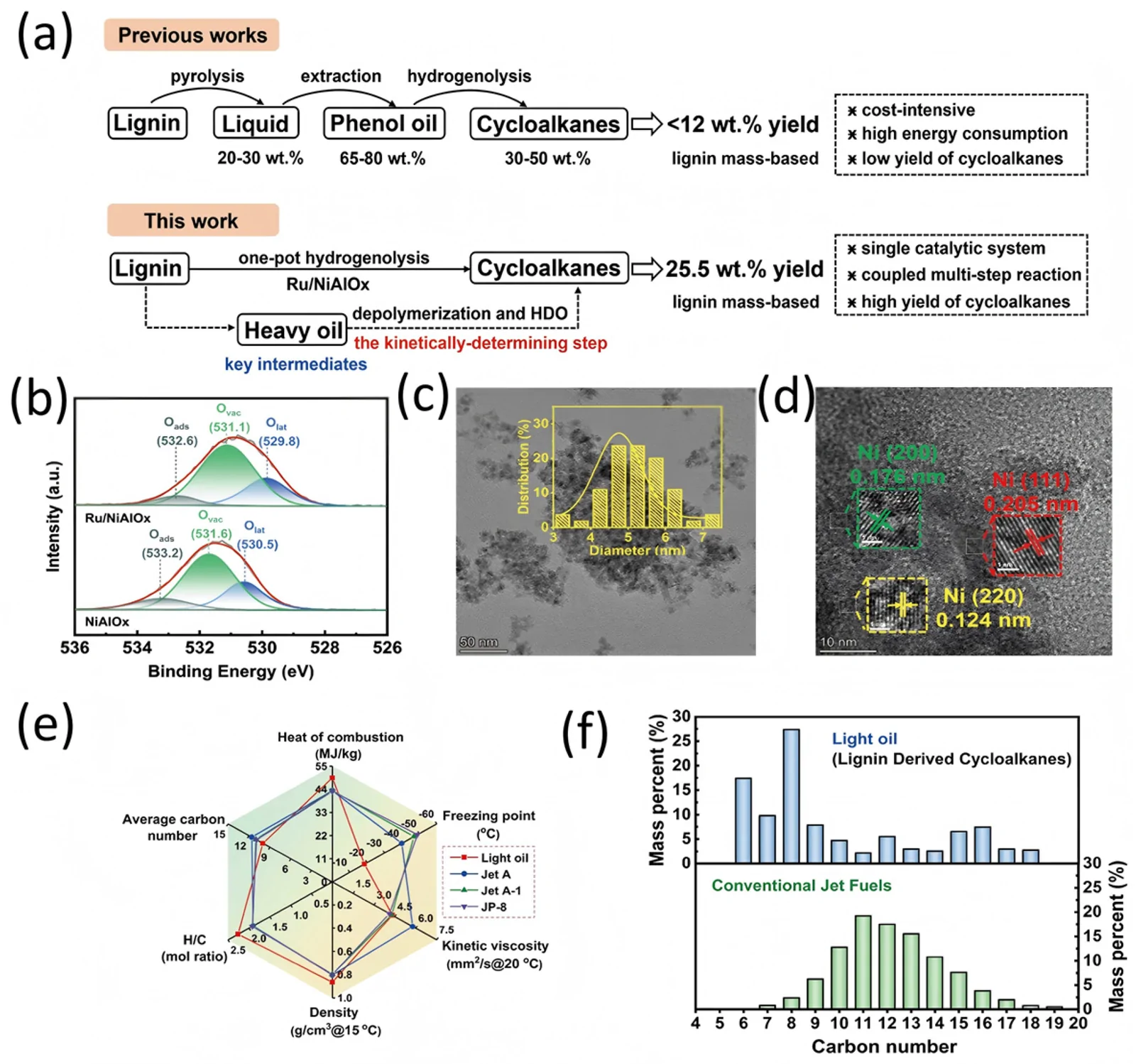
(**a**) Comparative evaluation of procedures and critical analysis of relative merits and limitations between the previous works and this work. (**b**) O 1s XPS spectrum of Ru/NiAlO*_x_*. (**c**,**d**) HRTEM image of Ru/NiAlO*_x_*. (**e**) Comparison of fuel properties in lignin-derived light oil and commercial/military jet fuels. (**f**) Carbon distributions of light oil and CJFs. Reproduced with permission from [231]; published by American Chemical Society, 2025.

In contrast, the one-pot approach simplifies the process flow and improves the yield of target products by optimizing the cascade reactions while minimizing side reactions, thereby enhancing both cost-effectiveness and energy efficiency [110,230]. The integration of direct catalytic depolymerization of lignin with HDO in a single reactor represents a viable route for producing stable cycloalkanes. Mao et al. [231] constructed a hydrotalcite-derived bimetallic Ru/NiAlO*_x_* catalyst for the one-pot conversion of lignin to cycloalkane-rich SAF precursors (Figure 19a). This catalyst promoted the cleavage and HDO of phenolic oligomers in lignin through metal–metal and metal–support synergy: electron transfer from Ru to Ni nanoparticles increased the electron density of Ni sites, enhancing H_2_ adsorption and activation; the generated active hydrogen species further migrated to the oxygen vacancies at the metal-oxide interface, promoting the adsorption of oxygenated oligomers and C-O bond cleavage (Figure 19b). Meanwhile, the incorporation of Ru suppressed the agglomeration of Ni particles, and the smaller particle size (4.2 nm, Figure 19c,d) along with a high specific surface area (197.53 m^2^/g) provided abundant active centers and sufficient reaction space for the efficient HDO of oxygenated intermediates. Compared with monometallic catalysts and physically mixed systems, Ru/NiAlO*_x_* exhibited a higher cycloalkane yield, indicating that the electronic coupling at the Ru-Ni interface and oxygen vacancy regulation were key factors for enhancing the cascade reaction efficiency. Over five consecutive cycles, this catalyst achieved a lignin deoxygenation degree of 78.5% and maintained a cycloalkane yield of 31.9–36.6 C% (Figure 19e,f). The results further demonstrated that the resulting cycloalkanes possessed fuel properties comparable to those of conventional jet fuel, including a high heating value (49.4 MJ/kg), an optimal H/C ratio (2.3 mol/mol), suitable kinematic viscosity (4.3 mm^2^/s @ 20 °C), and appropriate density (0.865 g/cm^3^ @ 15 °C). This work demonstrates that rational bifunctional catalyst design, through multi-active-site synergy, enables the efficient one-pot conversion of waste lignin into cycloalkane-type SAF components.

However, the sources and extraction processes of industrial lignin vary significantly, leading to considerable fluctuations in molecular weight, solubility, and impurity content. Such variability may give rise to issues including limited mass transfer of substrates, catalyst surface coking, and even reactor blockage during the actual cascade conversion process [232]. Therefore, when designing nanocatalysts for cascade conversion, it is essential to take full account of the complex composition of industrial lignin and its compatibility with the reaction medium, and to improve the accessibility of macromolecular substrates through strategies such as pore structure optimization and surface polarity modulation [233,234]. Meanwhile, a rational balance between acid and metal functionalities is critical for achieving in situ synergy between depolymerization and HDO while suppressing intermediate repolymerization and condensation; otherwise, coking, carbon loss, and rapid catalyst deactivation may occur [235].

## 4. Conclusions

This review systematically summarizes recent research progress on a series of nanocatalysts for the HDO of lignin to produce jet-fuel-range cycloalkanes and aromatics. Focusing on catalyst composition, structural design, and reaction pathway regulation, the review highlights the performance characteristics and mechanistic roles of molybdenum-based catalysts, nickel-based catalysts, bimetallic catalysts, bifunctional catalysts, and single-atom catalysts. The core scientific challenge in HDO lies in how to achieve selective cleavage of C-O bonds while precisely controlling the degree of aromatic ring hydrogenation, so as to meet the dual requirements of jet fuel for both aromatics (8–25%) and cycloalkanes. Addressing this challenge, existing studies have developed distinctive design strategies based on different catalyst types: molybdenum-based catalysts leverage MoS_2_ edge-site engineering and oxygen vacancy regulation, utilizing the tunability of layered structures to achieve targeted deoxygenation under mild conditions; nickel-based catalysts focus on particle size control and hydrogen spillover/transfer strategies to enhance low-temperature activity and C-C/C-O cleavage capability; bimetallic catalysts achieve optimized deoxygenation selectivity through geometric, electronic, and synergistic effects; bifunctional catalysts realize cascade synergy through the proximity effect between acid and metal sites; and single-atom catalysts enable precise manipulation of product selectivity through fine-tuning of coordination environment and charge density. These strategies, operating at the three levels of geometric structure, electronic density, and spatial organization of active sites, collectively provide a systematic catalyst design framework for resolving the trade-off between selective C-O bond cleavage and competing aromatic ring hydrogenation.

In summary, the precise design of nanocatalysts has become a core driving force for promoting the targeted conversion of lignin into jet fuel components (aromatics and cycloalkanes). Current research has extended from model compounds to real lignin feedstocks, and substantial progress has been made in terms of mild reaction conditions, high selectivity, and catalyst stability. Nevertheless, several challenges remain for practical applications: 1. The poisoning mechanisms of impurities (sulfur, water, inorganic salts) in real lignin toward the structure and long-term stability of catalyst active sites remain unclear, lacking support from in situ dynamic characterization. 2. Catalyst performance evaluation still relies heavily on model compounds [86,236,237]; the complex structure of lignin itself and the structural changes introduced during extraction make it difficult to accurately establish structure–activity relationships on actual substrates. 3. Simultaneously meeting the strict specifications of jet fuel regarding carbon number distribution, aromatics content, and cycloalkane proportion imposes differentiated and even competing demands on the geometric configuration and electronic density of catalyst active sites; how to achieve coordinated regulation of multiple selectivities within a single catalytic system remains a major challenge. 4. The catalyst-to-lignin ratio remains relatively high, resulting in low reaction efficiency, and there is an urgent need for breakthroughs in controllable synthesis and regenerating technologies, from laboratory scale to industrial-scale production. 5. The efficiency of hydrogen activation and transfer directly influences deoxygenation pathway selectivity and hydrogen consumption costs; the balance between hydrogen spillover capability and hydrogen dissociation/hydrogenation activity of the catalyst still needs further optimization. These metrics will ultimately be necessary to compare laboratory-scale catalyst performance on a consistent industrial SAF basis. In view of these challenges, the authors believe that future research at the catalyst level should focus on the following aspects: 1. combining in situ/operando characterization techniques (e.g., XPS, XAFS, electron microscopy) to reveal impurity-induced deactivation mechanisms, and designing poisoning-resistant catalytic systems accordingly; 2. establishing a stepwise catalyst evaluation system from model molecules to real lignin oil, and developing structure–activity correlation models through machine learning; 3. achieving synergistic optimization of aromatic ring hydrogenation and C-O bond cleavage, thereby meeting jet fuel composition specifications, through strategies such as spatial separation of active sites (e.g., the proximity effect in bifunctional catalysts), charge density modulation (e.g., single-atom coordination environment design), or kinetic partitioning of reaction pathways (e.g., cascade catalysis); 4. developing scalable catalyst synthesis routes (e.g., continuous flow synthesis, template-free green synthesis) and mild regeneration processes; 5. exploring alternative hydrogen source strategies such as catalytic transfer hydrogenation and photo/electrocatalytic in situ hydrogen production, while optimizing hydrogen spillover performance to reduce the hydrogen-to-oil ratio and thereby lower hydrogen source costs; 6. in addition, to enable meaningful comparison of laboratory-scale catalysts from an industrial SAF perspective, future studies should systematically report long-term continuous-flow testing, catalyst productivity, regeneration efficiency, and hydrogen consumption, as these metrics are currently scarce but ultimately indispensable. Techno-economic analysis (TEA) is therefore essential to determine whether catalyst-level gains in the comprehensive metrics above can be translated into cost-competitive, industrially viable lignin-derived SAF production [20]. Achieving these breakthroughs will rely on the synergistic application of multi-scale research methodologies and interdisciplinary approaches such as artificial intelligence, so as to advance the industrialization of lignin-based sustainable aviation fuel.

## Figures and Tables

**Table 2:** A summary of the recent literature on the application of nickel-based catalysts in the catalytic HDO of lignin.

Year	Catalyst	Substrate	Major Product	Catalyst to Biomass Ratio (C:B)	Size (nm)	Reaction Conditions				Conv. (%)	Sel. (%)	Ref.
						T (°C)	P (MPa)	Time (h)	Solvent			
2025	Ni@AlPO_4_/Al_2_O_3_	Guaiacol	Cyclohexane	1:2.5	7.1	230	1	2	*n*-Hexane	99.1	92.9	[112]
2022	Ni_2_P-Al_2_O_3_	Guaiacol	Cyclohexane	1:2.5	8.4	250	5	3	*n*-Hexane	100	94.5	[113]
2026	NiNPs-SAs/NC	PPE ^a^	Ethylbenzene	1:1.25	n.r.	200	none	3	Isopropanol	99.90	86.3	[114]

^a^ 2–2-phenoxy-1-phenylethane. n.r.: not reported.

**Table 3:** A summary of the recent literature on the application of bimetal catalysts in the catalytic HDO of lignin.

Year	Catalyst	Substrate	Major Product	Catalyst to Biomass Ratio (C:B)	Size (nm)	Reaction Conditions				Conv. (%)	Sel. (%)	Ref.
						T (°C)	P (MPa)	Time (h)	Solvent			
2020	RuNi/SiO_2_-ZrO_2_	Guaiacol	Hydrocarbons	1:2	n.r.	260	1	4	Octane	100	99.9	[132]
2021	Ni-Mo-0.1	Guaiacol	Cyclohexane	1:3.7	7.0	230	3	1	*n*-Decane	100	98.3	[133]
2025	Pd_1.7_-Pt_0.3_/NiO	Guaiacol	Cyclohexane	1	3.35	210	0.1	6	*n*-tetradecane	99.7	97.2	[134]

n.r.: not reported.

**Table 4:** A summary of recent literature on the application of bifunctional catalysts in the catalytic HDO of lignin.

Year	Catalyst	Substrate	Major Product	Catalyst to Biomass Ratio (C:B)	Size (nm)	Reaction Conditions				Conv. (%)	Sel. (%)	Ref.
						T (°C)	P (MPa)	Time (h)	Solvent			
2023	Ni_13%_/HZ-5(25)	Oxydibenzene	Cyclohexane	1:2	5	160	5	2	*n*-Hexane	79.90	80.80	[144]
2024	Ru/HZSM-5-P	Guaiacol	Cyclohexane	1	11.9	160	2	2	*n*-Hexane	99.9	99.9	[145]
2021	Ru/TiO_2_-CeO_2_	Guaiacol	Cyclohexane	1:2	n.r.	230	1	4	Octane	99.9	99.9	[146]
2025	Ni/Ti_0.5_Zr_0.5_O_2_	Anisole	Cyclohexane	1:2	n.r.	230	4	3	Decane	100	94.3	[147]
2022	Ru/C-HPW	Guaiacol	Cycloalkanes	1:2	n.r.	200	1	2	Octane	100	98.60	[137]
2020	SiW_12_-Pt/C	Phenol	Benzene+ Cyclohexane	1:20	n.r.	85	0.064	0.5	Water	100	90.89	[148]
		Guaiacol				95	0.042	0.75	Water	100	83.4	[148]
2024	Ni/NOAC	Guaiacol	Benzene+ Cyclohexane	1	8.6	300	2.5	5	Water and decalin	100	17.1 ^a^	[149]
2021	Pt/UiO-66-20def	Anisole	Benzene+ Cyclohexane	N/A	3.5	270	1	3	Decane	86%	84	[150]

N/A: not applicable. ^a^: HDO efficiency.

**Table 5:** A summary of recent literature on the application of single-atom catalysts in the catalytic HDO of lignin.

Year	Catalyst	Substrate	Major Product	Catalyst to Biomass Ratio (C:B)	Reaction Conditions				Conv. (%)	Sel. (%)	Ref.
					T (°C)	P (MPa)	Time (h)	Solvent			
2025	Pt_1_/NiAl-IMC	4-Propyl guaiacol	Propyl cyclohexane	1:2.5	245	2	2	*n*-Isooctane	100	96	[208]
2024	Pt_0.4_/CsPW-H_2_	4-Propyl guaiacol	Propyl cyclohexane	1:3.3	150	2	4	*n*-Dodecane	98	91.60	[209]
2024	Ni_1_/β-Mo_2_C	Dihydroeugenol	Propyl cyclohexane	1:1.66	250	4	2	*n*-Undecane	100	100	[111]
2025	Mo/NiFe_71_-V	Guaiacol	Cyclohexane	1:2	240	4	3	*n*-Hexane	100	99.5	[210]

## Data Availability

No new data were created or analyzed in this study. Data sharing is not applicable to this article.
